# Oenological traits of *Lachancea thermotolerans* show signs of domestication and allopatric differentiation

**DOI:** 10.1038/s41598-018-33105-7

**Published:** 2018-10-04

**Authors:** Ana Hranilovic, Joanna M. Gambetta, Leigh Schmidtke, Paul K. Boss, Paul R. Grbin, Isabelle Masneuf-Pomarede, Marina Bely, Warren Albertin, Vladimir Jiranek

**Affiliations:** 1 The Australian Research Council Training Centre for Innovative Wine Production, Adelaide, SA Australia; 2 Department of Wine and Food Science, The University of Adelaide, Adelaide, SA Australia; 3 National Wine and Grape Industry Centre, School of Agricultural and Wine Science, Charles Sturt University, Wagga Wagga, NSW Australia; 4 CSIRO Agriculture and Food, Adelaide, SA Australia; 5 Unité de recherche Œnologie, Institut de la Science de la Vigne et du Vin, University of Bordeaux, Villenave d’Ornon, France; 6 Bordeaux Sciences Agro, Gradignan, France; 7 ENSCBP, Bordeaux INP, Pessac, France

## Abstract

The yeast *Lachancea thermotolerans* (previously *Kluyveromyces thermotolerans*) is a species of large, yet underexplored, oenological potential. This study delivers comprehensive oenological phenomes of 94 *L. thermotolerans* strains obtained from diverse ecological niches worldwide, classified in nine genetic groups based on their pre-determined microsatellite genotypes. The strains and the genetic groups were compared for their alcoholic fermentation performance, production of primary and secondary metabolites and pH modulation in Chardonnay grape juice fermentations. The common oenological features of *L. thermotolerans* strains were their glucophilic character, relatively extensive fermentation ability, low production of acetic acid and the formation of lactic acid, which significantly affected the pH of the wines. An untargeted analysis of volatile compounds, used for the first time in a population-scale phenotyping of a non-*Saccharomyces* yeast, revealed that 58 out of 90 volatiles were affected at an *L. thermotolerans* strain level. Besides the remarkable extent of intra-specific diversity, our results confirmed the distinct phenotypic performance of *L. thermotolerans* genetic groups. Together, these observations provide further support for the occurrence of domestication events and allopatric differentiation in *L. thermotolerans* population.

## Introduction

The largely untapped biotechnological potential of yeasts other than *Saccharomyces cerevisiae* is triggering rising scientific interest. One remarkable example is *Lachancea thermotolerans*, a ubiquitous species occupying a range of anthropic and wild habitats that cover a large geographic span^1–4^. In particular, this yeast is a common constituent of grape/wine microbiota^5,6^, and has thus been explored for its application in oenology. Indeed, multiple studies have evaluated the oenological performance of *L. thermotolerans* isolates^7–10^ delivering conclusive results; *L. thermotolerans* does not impart any obvious faults to the wine, rather, it can positively affect its chemical and sensory profile.

In oenological environments, *L. thermotolerans* is a relatively robust fermenter, depending on the strain and physiochemical conditions, capable of achieving up to 13.6% (v/v) ethanol^11^. As typical for non-*Saccharomyces* yeasts, *L. thermotolerans* pure cultures cannot ‘complete’ wine fermentation (i.e. deplete all sugars), and therefore require sequential or simultaneous addition of another co-starter, generally an *S. cerevisiae* strain^5^. Due to the antagonistic activity of *S. cerevisiae* towards *L. thermotolerans*, mediated by mechanisms of cell-cell contact and secretion of antimicrobial peptides^12^, the outcomes of such co-fermentations are inoculation-dependant. The initial absence and/or lower inoculation densities of *S. cerevisiae* allows for the prolonged persistence and, in turn, greater metabolic contribution, of *L. thermotolerans* strains^9,13,14^.

The major metabolic contribution of *L. thermotolerans* is lactic acid production concomitant to alcoholic fermentation^5,7–9^. The maximum reported lactic acid concentrations formed during *L. thermotolerans* fermentation exceed 16 g/L^15^, thus representing orders of magnitude that are unique among any other non-genetically modified yeasts^16,17^. *S. cerevisiae* wildtype strains, by comparison, in similar conditions normally produce only <0.4 g/L lactate^16,17^. The resultant acidification is considered to positively affect wine microbial stability and organoleptic balance, while alleviating the need for external inputs (e.g. tartaric acid) commonly used to acidify grapes from warmer climates/vintages^7,10,18^. Another common characteristic of such grapes is excessive sugar accumulation, leading to undesirably high ethanol concentrations in wines^19^. Several studies reported significantly lower ethanol contents in co-fermentations with *L. thermotolerans* and *S. cerevisiae* that ranged between 0.2% and 0.9% (v/v) less than their respective *S. cerevisiae* monoculture controls^9,10,14,20^, thus highlighting the potential of *L. thermotolerans* in production of lower-alcohol wines. Other beneficial/non-detrimental compositional alterations reported in *L. thermotolerans* treatments include increases in glycerol concentration^9,10,13,14^, decreases in acetate content^8–10,13,14^, partial degradation of malate^21–23^ and modulations of both grape- and yeast-derived volatile compounds in wines^10,13,14,22–25^.

The extent of intra-specific variability in traits of oenological importance among *L. thermotolerans* strains, however, remains elusive as the previous studies examined only a limited number of strains and/or metabolites, and employed different culture conditions and analytical techniques. Conversely, more insight is available into the genetic diversity of the *L. thermotolerans* population^2,4^. In our recent work we developed a 14-microsatellite genotyping method to study the relationship between 172 isolates from diverse habitats worldwide^4^. The natural isolates were grouped based on their geographic origin, whereas the genetic proximity of isolates from anthropic, in particular oenological environments, suggested domestication events within the species. Plate-based growth assays using different carbon substrates and physicochemical conditions provided further support for the observed clustering^4^. To determine whether, and to what extent*, L. thermotolerans* strains differ in oenologically-relevant traits, and harbour signatures of domestication and/or local divergence, we hereby report a comprehensive phenotypic characterisation of 94 previously genotyped strains in *Vitis vinifera* cv. Chardonnay fermentations.

## Results

### Fermentation performance of *L. thermotolerans* strains

The tested strains, obtained from diverse ecological niches worldwide, were classified into nine genetic groups (Supplementary Fig. S1; Table S1) as determined by microsatellite profiling^4^. Based on the isolation location and/or niche of their constituents, the genetic groups were considered as ‘wild’ (‘Americas’, ‘Canada trees’, ‘Hawaii/California’, ‘Other’), ‘domestic’ (‘Domestic 1’, ‘Domestic 2’) and ‘mixed’ (‘Mix Eastern Europe’, ‘Mix Europe/North America’, ‘Europe oak/France grapes’), with a balanced number of strains representing the three classes. The ‘wild’ groups were comprised predominantly of natural isolates, clustered together based on their geographic origin, while the ‘domestic’ groups harboured isolates from anthropic, mainly oenological, environments. The remaining groups were ‘mixed’ with regards the substrate of isolation and/or geographic location of the strains. The strains and the genetic groups were compared for the microbial growth and sugar consumption kinetics, final production of primary and secondary metabolites and pH modulation in 25 mL Chardonnay grape juice fermentations (Supplementary Fig. S2).

**Figure 1:**
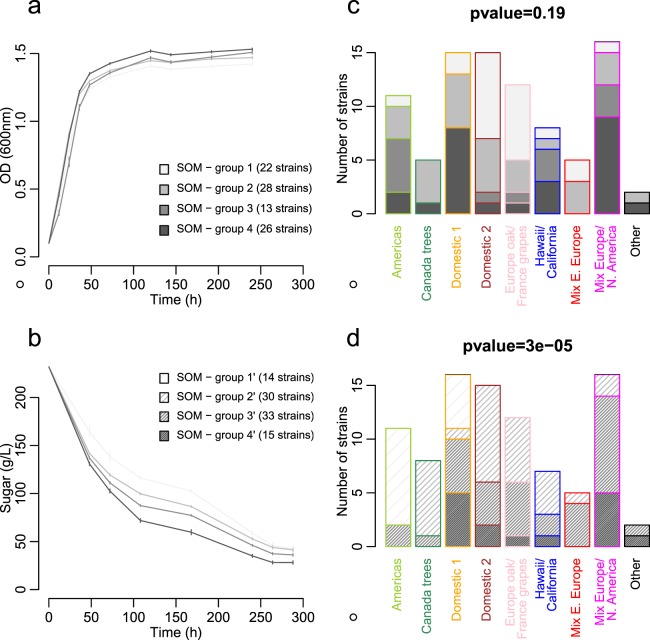
Self-Organizing Maps of growth and sugar consumption kinetics in *L. thermotolerans* fermentations. SOM analysis identified four clusters that best discriminate different genetic groups based on strain growth (**a**) and sugar consumption kinetics (**b**). Lines represent mean values and standard errors of SOM clusters. Within each genetic group, the number of strains belonging to different growth- and sugar consumption-related SOM clusters is represented in (**c**) and (**d**) barplots, respectively. Distribution of genetic groups within each SOM was determined by chi^2^ tests with the corresponding p-values noted in (**c**) and (**d**). Colour-coding represents different genetic groups, as per the legends in (**c**) and (**d**).

All strains were able to proliferate (Fig. 1a) and catabolise sugars (Fig. 1b) despite the extreme conditions inherent to winemaking (e.g. high sugar content, limited assimilable nitrogen, rapid oxygen depletion). Spectrophotometric growth monitoring was not possible for three strains (LL12-031, LL12-056 and UWOPS 79-110) due to the pronounced flocculation. The frequent monitoring of microbial growth (OD_600_) and sugar consumption allowed for the fermentation kinetics to be subjected to Self-Organizing Map (SOM) analysis. SOM of population growth that best explained the differentiation among genetic groups contained four clusters (Fig. 1a). However, the distribution of the different genetic groups amongst SOM clusters was not significant (Fig. 1c; chi^2^ test p-value = 0.19). Conversely, SOM of sugar consumption kinetics resolved four clusters, which corresponded to low (group 1′), medium-low (group 2′), medium-quick (group 3′) and quick (group 4′) sugar consumption kinetics (Fig. 1b). The SOM with low fermentation kinetics (group 1′) contained 14 strains, i.e. nine ‘Americas’ and five ‘Domestic 1’ genotypes. Comparable number of strains displayed medium-low (group 2′; 30) and medium-quick (group 3′; 33) sugar consumption kinetics. These belonged to different genetic groups (Fig. 1d). In contrast, none of the 15 strains displaying quick sugar consumption kinetics (group 4′) were ‘Americas’ and ‘Canada trees’ strains. Disproportionate distribution of SOM clusters within each genetic group was confirmed by chi^2^ test (p-value = 3.10e-5), with an over-representation of low and medium-low fermenters in the ‘Americas’ and ‘Canada trees’ groups, and medium-quick and quick fermenters in ‘Mix Europe/North America’ (Fig. 1d).

### Phenotypic variation in main fermentation metabolites

The extent of final sugar consumption in Chardonnay grape juice fermentations ranged between 161.6 and 223.4 g/L (Table 1). All the strains displayed a glucophilic character, i.e. consumed more glucose than fructose, with variable residual glucose/fructose ratios (Table 1). The achieved ethanol concentrations varied between 7.3 and 10.6% (v/v), however, strains did not significantly differ in their ethanol production capacity (Table 1). Strain 72–132 exhibited extreme glycerol production levels (8.0 g/L), while for most other strains glycerol concentrations and yields were more closely distributed around the mean values (Table 1). Interestingly, the concentrations and yields of glycerol, generally the second most abundant wine fermentation metabolite after ethanol, were lower than those of lactate in 48 strains. The highest lactic acid concentration (12.0 g/L) was produced by 68–140 (Table 1). The same strain consumed the highest concentration of malate, i.e. 0.8 g/L (Table 1). While partial degradation of malate was observed in most treatments, some strains led to an increase in malate of up to 0.3 g/L (LL12_056). In a winemaking context, acetic acid concentrations and yields in all *L. thermotolerans* fermentations were relatively low, and pyruvic acid concentrations ranged between 13 and 78 mg/L (Table 1). A decrease in pH from the initial value of 3.5 was observed in the majority of fermentations (i.e. 68/94). The largest pH drop, that of 0.34 units, observed in strain 68–140 (Table 1). Conversely, a minority of strains elicited de-acidification, with DBVPG 3466 having the highest pH value of 3.81 (Table 1).

**Table 1:** Analytical properties of Chardonnay wines fermented by different *L. thermotolerans* strains.

Parameter	Minimum^a^		Maximum^a^		Mean^b^
Consumed sugar (g/L)	161.6 ± 1.4	(DBVPG 3464)	223.4 ± 2.9	(NZ156)	199.6 ± 11.4
Glucose (g/L)	3.7 ± 0.5	(DBVPG 3466)	36.2 ± 0.7	(DBVPG 3464)	13.6 ± 6.2
Fructose (g/L)	8.9 ± 1.3	(NZ156)	39.1 ± 1.6	(72–132)	23.2 ± 6.0
G/F^c^	0.16 ± 0.02	DBVPG 3464	0.94 ± 0.01	UWOPS 94–426.2	0.57 ± 0.16
*Ethanol (% v/v)*	7.3 ± 0.7	(72–132)	10.6 ± 0.7	(CBS 2907)	9.3 ± 0.9
*Ethanol yield* ^d^ *(g/g)*	0.34 ± 0.04	(8/Z-1)	0.40 ± 0.03	(DBVPG 3464)	0.37 ± 0.03
Glycerol (g/L)	3.9 ± 0.1	(DBVPG 3464)	8.0 ± 0.2	(72–132)	5.4 ± 0.6
Glycerol yield^d^ (g/g)	0.0205 ± 0.0004	(CBS 137)	0.0478 ± 0.0002	(72–132)	0.0274 ± 0.0039
Lactic acid (g/L)	1.8 ± 0.2	(JCB1)	12.0 ± 0.2	(68–140)	5.8 ± 2.3
Lactate yield^d^ (g/g)	0.0086 ± 0.0011	(JCB1)	0.0658 ± 0.0018	(68–140)	0.0291 ± 0.0119
Acetic acid (g/L)	0.06 ± 0.01	(72–137)	0.32 ± 0.01	(UWOPS 85–51.1)	0.20 ± 0.05
Acetate yield^d^ (mg/g)	0.30 ± 0.05	(72–137)	1.53 ± 0.03	(UWOPS 85–51.1)	0.98 ± 0.24
FP^e^	0.0067 ± 0.0008	72–137	0.0327 ± 0.0040	LL13–189	0.0213 ± 0.0056
Pyruvic acid (mg/L)	13 ± 1	(MUCL 31342)	78 ± 3	(9/1/Z-4)	44 ± 14
Malic acid (g/L)	3.0 ± 0.1	(68–140)	4.1 ± 0.2	(LL12_056)	3.6 ± 0.3
pH	3.16 ± 0.03	(68–140)	3.81 ± 0.13	(DBVPG 3466)	3.44 ± 0.03

^a^Minimum and maximum values (means of triplicates ± standard errors) associated with the strains in the brackets; ^b^Mean values of all observations (±standard errors); ^c^G/F - measure of fructophilicity defined as residual glucose and fructose ratio; ^d^Yields were calculated from concentrations of respective metabolites and consumed sugar; ^e^FP - fermentation purity defined as a ratio of acetic acid and ethanol. Chardonnay grape juice contained 236.4 g/L sugar (1:1 mix of glucose and fructose), 3.8 g/L malic acid and pH 3.5. Differences for parameters in italics were not significant (KW test, p-values > 0.05).

**Figure 2:**
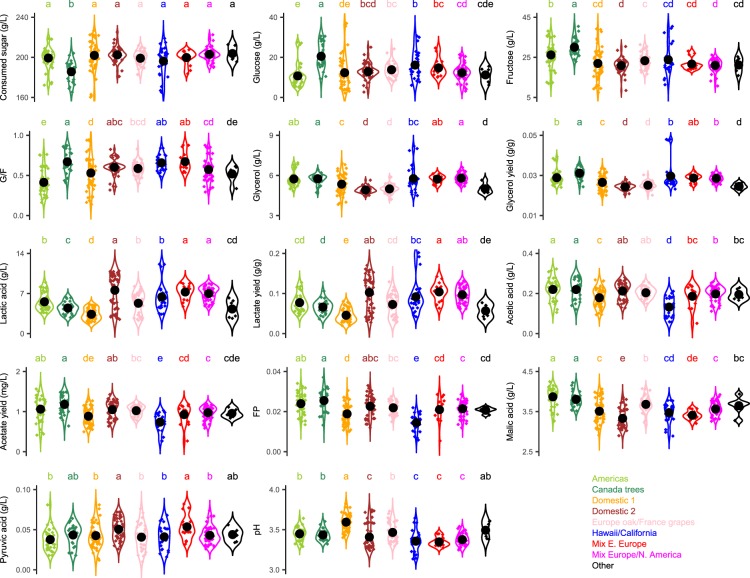
Violin plots for the selected metabolic traits. For each genetic group, numeric values are represented as diamonds, the corresponding probability densities are represented as plain traits, mean and standard error are represented by black circles and segments, respectively (*ggplot2* package, R). Top letters represent significance groups as defined by Kruskal-Wallis test (*agricolae* package, p-value < 0.05 after Benjamini & Hochberg adjustment for multiple comparisons).

The parameters showing a significant strain effect (Table 1) were further compared at a genetic group level (Fig. 2). ‘Canada trees’ strains were characterised by the lowest extent of sugar consumption as a result of the highest concentrations of glucose and fructose alike (Fig. 2). The ‘Americas’ strains, i.e. another group comprised of natural isolates, were capable of extensive glucose consumption, however, their residual fructose content was higher than in any other group except ‘Canada trees’ (Fig. 2). Consequently, the fructophilicity (G/F) of ‘Americas’ strains was low. The concentrations and yields of glycerol were generally higher for most wild and mixed groups, than for the domestic ones (Fig. 2). ‘Domestic 1’ strains had lower concentrations and yields of lactate and the higher pH values than all other groups except ‘Other’ (Fig. 2). Lactate production was the highest in ‘Domestic 2’, ‘Mix Eastern Europe’ and ‘Mix Europe/North America’ groups. Acetic acid was the lowest in strains belonging to ‘Hawaii/California’ group, and their acetate yields and fermentation purity (FP) were also low (Fig. 2). The levels of malic acid in ‘Canada trees’ and ‘Americas’ wines were higher than in all other groups, and pyruvate concentrations were higher in ‘Domestic 2’ and ‘Mix Eastern Europe’ than in most other groups (Fig. 2).

### Volatile metabolome of *L. thermotolerans* wines

**Figure 3:**
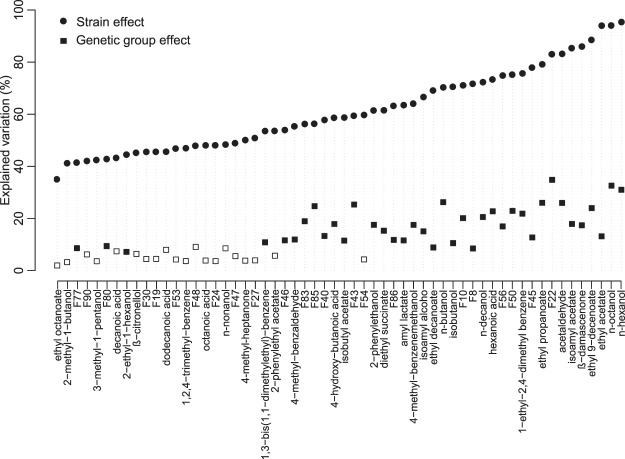
Percentage of variation in volatile compounds explained by either strain or genetic group effect. Only 58 compounds with a significant (p-value < 0.05) strain effect are shown. For the genetic group effect, white squares represent no significant p-values (>0.05) and coloured squares indicate significant p-values (<0.05).

The obtained Chardonnay wines were also analysed for their volatile composition. Out of 90 analysed volatiles, 58 compounds were affected at a strain level (Fig. 3; Supplementary Table S2). The majority of these compounds (35/58) were successfully identified in the NIST database via corresponding mass spectra, Kovats’ RI indices and, when available, comparison with pure compounds (Supplementary Table S2). The remaining 23 compounds were unidentified (unknown; 23/58). The identified compounds included higher alcohols (12/58), with the representatives of C_6_ (n-hexanol), aryl (2-phenylethanol and 4-methyl-benzenemethanol), branched (isobutanol, isoamyl alcohol, 2-methyl-1-butanol, 3-methyl-1-pentanol, 2-ethyl-hexanol) and non-branched compounds (n-butanol, n-nonanol, n-octanol and n-decanol). A comparable number of ethyl esters was detected (10/58). These included ethyl esters (ethyl propanoate, ethyl octanoate, ethyl decanoate, ethyl 9-decenoate, diethyl succinate), acetates (ethyl acetate, isobutyl acetate, isoamyl acetate and 2-phenylethyl acetate), and a lactate (amyl lactate). Five acids also significantly differed between the strains (5/58; 4-hydroxy-butanoic, hexanoic, octanoic, decanoic and dodecanoic acid). The remaining compounds were classified as aromatic compounds (3/58; 1-ethyl-2,4-dimethyl benzene, 1,2,4-trimethylbenzene and 1,3-bis(1,1-dimethylethyl) benzene), aldehydes (2/58; acetaldehyde and 4-methyl-benzaldehyde), a ketone (1/58; 4-methyl-2-heptanone), a norisoprenoid (1/58; ß-damascenone) and a terpenol (1/58; β-citronellol).

n-Hexanol and n-octanol were the volatiles that displayed the largest strain effect and, after F22, genetic group effect (Fig. 3). These two higher alcohols followed the same trend; their content was lower in ‘Domestic 1’ wines than those from any of the remaining groups except ‘Other’, and high in ‘Mix Eastern Europe’, ‘Mix Europe/North America’ and ‘Domestic 2’ groups (Supplementary Fig. S3). The ‘Domestic 1’ group also produced less n-butanol and several unidentified compounds (e.g. F43 and F50) compared to most other groups. Conversely, F22 and ß-damascenone were relatively high in the ‘Domestic 1’ group, as were isobutanol, F10 and F56. The ‘Americas’ strains produced high levels of acetaldehyde, ethyl acetate, ethyl propanoate, isoamyl acetate, isoamyl alcohol and ethyl 9-decenoate (Supplementary Fig. S3). Ethyl 9-decenoate was similarly high in ‘Domestic 2’ and ‘Mix Eastern Europe’ wines, which were also characterised by an increase in ethyl decanoate and F86 (Supplementary Fig. S3). The ‘Canada trees’ group was related to a low production of F85, 2-phenylethanol, isobutyl acetate, diethyl succinate, 4-methyl-benzaldehyde and 1,3-bis(1,1-dimethylethyl) benzene, and overproduction of 1-ethyl-2,4-dimethylbenzene (Supplementary Fig. S3). The latter aromatic compound was, in addition to F8, F40 and F46, particularly low in the ‘Hawaii/California’ group (Supplementary Fig. S3). The ‘Mix Europe/North America’ strains produced high levels of 4-methylbenzene methanol and F43 (Supplementary Fig. S3). Hexanoic acid and F83 were also high in this group, as well as in ‘Mix Eastern Europe’, ‘Americas’ and ‘Canada trees’, while 4-hydroxybutanoic acid was low in all these groups but ‘Canada trees’ (Supplementary Fig. S3). The ‘Europe oak/France grapes’ strains yielded less n-decanol and amyl lactate than all groups but ‘Canada trees’ and/or ‘Other’ (Supplementary Fig. S3).

### Phenotypic differentiation of *L. thermotolerans* genetic groups

**Figure 4:**
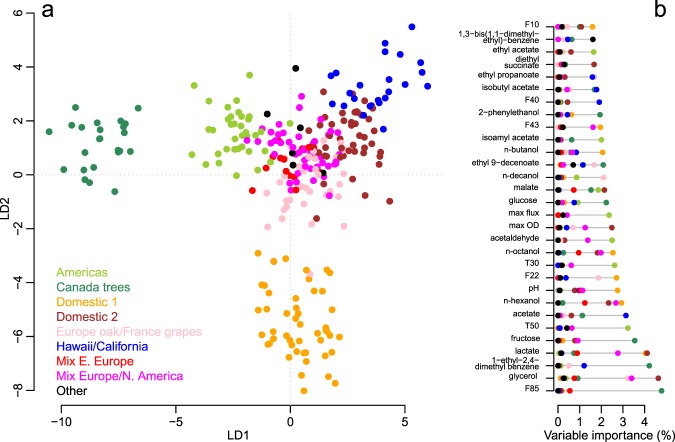
Linear discriminant analysis of nine genetic groups in *L. thermotolerans* based on 107 variables. LDA plot showing the first two axes, i.e. LD1 and LD2 (**a**). The importance of variables accounting for LDA was computed for each genetic group (**b**). Only the main 30 variables are shown; all 107 variables are in Supplementary Fig. S4.

To determine whether the obtained metabolic dataset discriminated the *L. thermotolerans* genetic groups, 107 variables were subjected to linear discriminant analysis (LDA). These analysed variables included fermentation kinetics parameters, concentrations of main metabolites, volatile compounds and pH. LDA revealed a clear separation of the ‘Canada trees’ and ‘Domestic 1’ groups from all remaining groups (Fig. 4a). Albeit less resolved, a suitable partitioning of strains belonging to the remaining genetic groups of strains was also obtained, and the co-localisation of ‘Mix Eastern Europe’ and ‘Mix Europe/North America’ groups was congruent with their genetic proximity^4^.

The ‘Canada trees’ group were differentiated from other groups based on the low production of the unknown volatile compounds F85, which represented the most important variable for discriminating genetic groups (Fig. 4b; Supplementary Fig. S4), as well as the high concentrations of 1-ethyl-2,4-dimethyl-benzene and both grape hexoses (Fig. 2; Supplementary Fig. S3). Low lactate, n-hexanol and n-octanol, and high pH and F22 were amongst the most important variables driving the separation of ‘Domestic 1’ strains (Fig. 2; Supplementary Fig. S3). Glycerol, overall ranked as the second most relevant variable for LDA, was of main importance for ‘Domestic 2’ group, as well ‘Mix Europe/North America’ and ‘Europe oak/France grapes’ (Fig. 4b). A similarly important metabolite for ‘Domestic 2’ and ‘Mix Europe/North America’ was high lactate, followed by low maximum ODs for the former group, and high n-hexanol and n-octanol for both groups (Supplementary Fig. S3). ‘Americas’ strains were discriminated based on the parameters related to their sugar consumption kinetics (i.e. high T50, T30 and max flux; Fig. 1b), and increased production of several volatile compounds (i.e. acetaldehyde, ethyl acetate and isoamyl acetate; Supplementary Fig. S3), and ‘Hawaii/California’ strain primarily due to their low acetic acid production (Fig. 2).

### Relationships between metabolites

**Figure 5:**
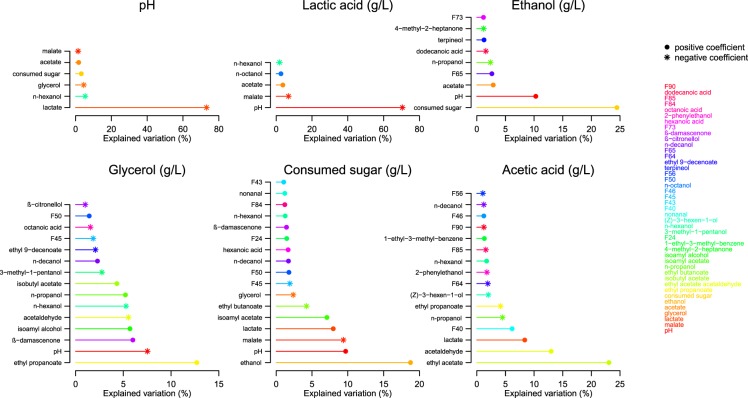
Relationships between metabolites of interest revealed by multiple linear regression analysis. Shown variables significantly (p-value < 0.01) explaining more than 1% of variation.

Multiple linear regression analysis was conducted to examine the relationships between the variables of interest (i.e. main fermentation products) and other metabolites as well as pH values. The analysis revealed that the most explanatory variable for pH was lactic acid, accounting for 73% of variation (Fig. 5). Likewise, pH explained 70% of variation in lactate concentrations of the wines. Besides sugar consumption (24% of explained variation), significant contributions to variation in ethanol formation were pH, acetic acid and several volatiles, which all had positive coefficients, except dodecanoic acid. The extent of sugar consumption was best explained by ethanol production (19% of explained variation), followed by pH, malate and lactate. After ethyl propanoate, pH was also the second most explanatory variable for obtained glycerol concentrations (8% of explained variation; negative coefficient). Several volatile compounds significantly accounted for both sugar consumption and glycerol production. Ethyl acetate and acetaldehyde together explained 38% of acetic acid concentration, and lactate contributed with an additional 8%.

**Figure 6:**
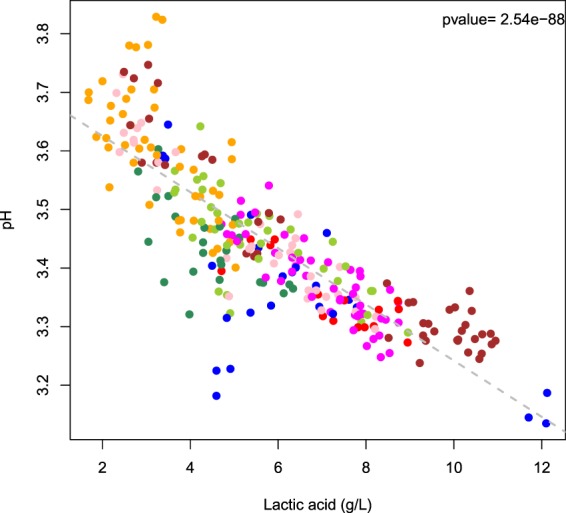
Correlation between lactic acid production and pH values in Chardonnay grape juice (pH 3.5) fermentations. Colour-coding represents *L. thermotolerans* genetic groups (as per Figs 1, 2, 4).

The correlation between pH and lactate was further confirmed by Spearman’s correlation test (Fig. 6), which was also used to assess the inter-relationships between consumed sugar concentrations and main fermentation by-products in *L. thermotolerans* fermentations, (i.e. ethanol, lactate, glycerol and acetate) within each genetic group (except ‘Other’) and the entire dataset (Supplementary Figs S5–S14). Interestingly, negative correlations were observed between the extent of sugar consumption and lactate production for certain genetic groups, but not globally (Supplementary Fig. S6). Similarly, correlations between lactate and glycerol were detected only within certain groups; positive within four and negative within one (‘Hawaii/California’; Supplementary Fig. S12). Both lactate and ethanol, and glycerol and ethanol showed weak negative correlations within the whole dataset, and for several genetic groups individually (Supplementary Figs S9, S10). No correlations whatsoever were detected for acetate and glycerol production, while acetate and lactate displayed weak positive correlations within the whole dataset and for two genetic groups (Supplementary Figs S13, S14).

## Discussion

There is a growing interest in the selection and characterisation of non-*Saccharomyces* yeasts to be used in winemaking to build ‘complexity’ and diversify styles. However, surprisingly few studies systematically explored the concepts of their intra-specific phenotypic variability^26–28^. To our knowledge, the scale and range of this work represents the broadest oenological characterisation of phenotypic variability within a population of a non-*Saccharomyces* yeast. It was designed to assess to what extent strains of *L. thermotolerans* vary in traits of key importance for fermentation outcome, i.e. microbial growth and sugar consumption kinetics, production of volatile and non-volatile metabolites and the resultant (de)acidification. The comprehensive phenotyping dataset was comprised of 114 measured/derived parameters for triplicate fermentations of 94 recently genotyped *L. thermotolerans* strains, resulting in over 32,100 individual data points. The tested strains were obtained from both natural and anthropic habitats, and distributed across the entire *L. thermotolerans* phylogenetic tree^4^ (Supplementary Fig. S1), so as to ascertain if the determined phenotypic variability is fully representative of the species.

The observed phenotypic variability was large and ethanol production was the only primary metabolic trait that did not differ between the strains, possibly due to analytical limitations. Similarly, differences in ethanol content alone, quantified with an equivalent HPLC method, were not detected between 72 *S. cerevisiae* strains in a population-scale phenotyping conducted under winemaking conditions by Camarasa *et al*.^29^, despite significant disparities in strain sugar consumption levels. Regardless, given that *S. cerevisiae* generally yields about 0.47 g of ethanol per 1 g of sugar^30^, the ethanol yields of *L. thermotolerans* strains determined here were altogether low (mean value 0.37 g/g; Table 1). Although the comparison of ethanol yields between different conditions and physiological stages is invalid, this attribute warrants further investigation for use of *L. thermotolerans* in the production of wines with lower-ethanol content, as observed elsewhere^9,10,14,20^.

In accord with previous reports^8,9,13,15^, our results confirmed that the common oenological features of *L. thermotolerans* strains are their glucophilic character, relatively extensive fermentation ability, albeit without ‘completion’, low production of acetate and formation of lactate. In contrast to previous findings, acidification was found for most, but not all, strains (Table 1). An increase in wine pH has, to our knowledge, thus far not been associated with *L. thermotolerans* fermentations despite the reports of partial degradation of malate^21^, as also witnessed in most of our treatments. Lactate concentrations were, by large, the most explanatory variable for the resultant pH modulation, as shown by multiple linear regression analyses (73% of explained variation; Fig. 5). Although the maximum concentrations of lactate achieved under current conditions (12 g/L) were lower than those from the literature (16.6 g/L)^15^, a seven-fold variation was detected for this trait (Table 1).

**Figure 7:**
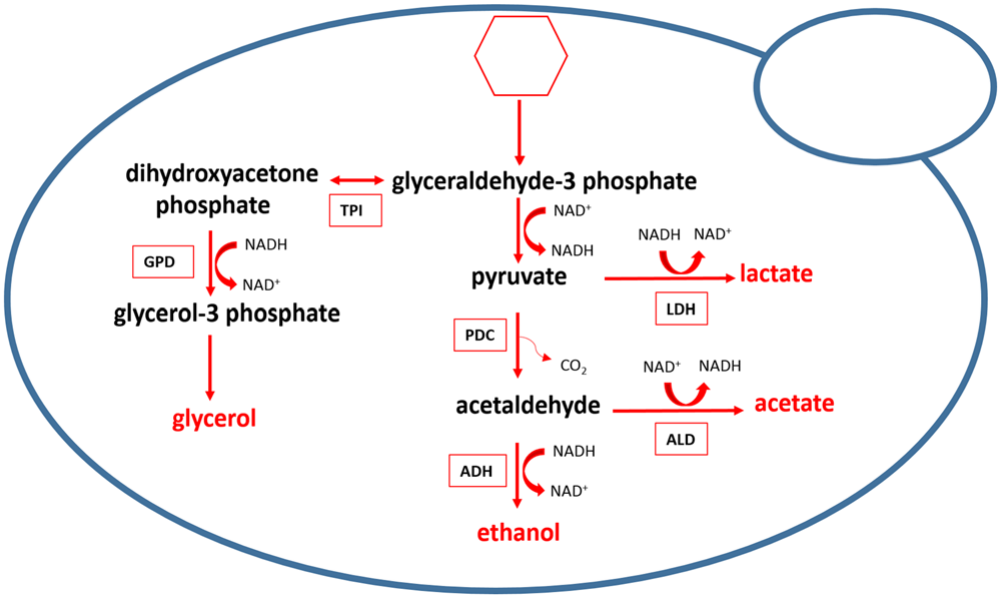
Production of the main fermentation (by)products in *L. thermotolerans*. Hexoses are metabolised via glycolysis to pyruvate through multiple enzymatic steps. Pyruvate is decarboxylated to acetaldehyde (pyruvate decarboxylase; PDC), which is further reduced to ethanol (alcohol dehydrogenase; ADH) or oxidised to acetate (aldehyde dehydrogenase; ALD). A proportion of pyruvate is converted to lactate (lactate dehydrogenase; LDH). Glycerol is produced via dihydroxyacetone phosphate (glycerol-3-phosphate dehydrogenase; GPD; triosephosphate isomerase; TPI) (adapted from Kegg Pathway Database^52^; http://www.genome.jp/kegg-bin/show_pathway?lth00010).

Lactic acid formation is, in fact, a metabolic hallmark of *L. thermotolerans*, but its physiological role and underlying molecular mechanisms remain poorly understood. From the literature, it is unclear whether in *L. thermotolerans*, as in lactic acid bacteria^16^, lactate formation via NAD-dependant lactate dehydrogenase (LDH) serves to re-plenish oxidised NAD^+^ depleted through glycolysis (Fig. 7). In yeasts, this is primarily achieved through formation of ethanol via the decarboxylation of pyruvate and the subsequent reduction of acetaldehyde, i.e. alcoholic fermentation^31^. In addition to osmoregulation, glycerol production in *S. cerevisiae* also serves as a redox valve to eliminate excess cytosolic NADH under anaerobic conditions and is coupled with acetic acid production^32,33^. Information of carbon flux and redox balance in *L. thermotolerans* is surprisingly scarce. Our data, nonetheless, highlighted several inter-relationships between metabolites of interest and pH values via multiple linear regression analyses and correlations. Most notable were the significant (and second largest) proportions of variation in ethanol and glycerol production explained by pH values, displaying positive and negative coefficients, respectively (Fig. 5). Moreover, negative (albeit weak) correlations between ethanol (despite the previously discussed analytical constraints) and both lactate and glycerol were observed, while lactate and glycerol correlated differently depending on the *L. thermotolerans* genetic group (no overall correlations, negative within four and positive within one genetic groups; Supplementary Figs S9, S10, S12). These observations potentially suggest that *L. thermotolerans* strains differ in their metabolic strategies to restore the NADH/NAD^+^ balance. Furthermore, the contribution of lactate, but not glycerol, towards variation in acetate (Fig. 5), and the absence of correlations between glycerol and acetate production (Supplementary Fig. S14) indicates that *L. thermotolerans*, as reported for some other non-*Saccharomyces* species such as *T. delbureckii*^34^, differ from *S. cerevisiae* in their metabolic link between glycerol and acetate production. Altogether, these findings invite further investigation of central carbon metabolism in *L. thermotolerans*, in particular the regulatory framework of redox balance, through studies purposely designed to quantify the microbial growth and evolution of metabolites in conjunction with transcriptomics.

In addition to the analysis of primary metabolites, this work, for the first time, implemented a metabolomics approach to study volatile footprints of a non-*Saccharomyces* yeast population. Recently, the volatile metabolome of a commercial *L. thermotolerans* strain was explored in the context of inter-specific comparison of several wine-associated non-*Saccharomyces* yeasts and a *S. cerevisiae* control, using a targeted approach either at an early fermentation stage^35^ or an untargeted approach in wines completed with sequentially inoculated *S. cerevisiae*^22,23^. This extensive comparison revealed that, within each matrix (i.e. Syrah and Sauvignon Blanc) and fermentation modality (i.e. pure culture and co-culture), the modulation of wine volatile profiles was species-dependant^22,23,35^. Our data show that a wide array of volatile compounds are also affected at an *L. thermotolerans* strain level (Fig. 3). The high-throughput nature of the applied methodology^36^ and an untargeted approach allowed us to study the volatile compounds that might have otherwise been overlooked, yet they significantly differentiated the strains and genetic groups of *L. thermotolerans*. The best examples are the unidentified compounds F22 and F85, which displayed the largest genetic group-effect (Fig. 3) and an importance for genetic group discrimination in LDA (Fig. 4), respectively. Besides the unidentified compounds, the majority of strain-affected volatile compounds represented the main constituents of the so-called secondary, fermentation-derived volatile aroma, i.e. esters and higher alcohols^37,38^. However, the effect on the primary aroma compounds was equally, if not more, pronounced, as some of the most strain-affected compounds were in fact grape-derived (i.e. n-hexanol, n-octanol, ß-damascenone)^39^. The observed variation in volatile composition of wines partially arises from the differential sugar consumption levels (Fig. 5) and, potentially, the matrix effect on the headspace partitioning of the aroma compounds^40^. However, inter-strain differences in mechanisms involved in the biosynthesis of volatile compounds, including enzymatic activities (e.g. esterase, glucosidase, acetyltransferase), amino acid metabolism and fatty acid synthesis require further investigation.

The genotyping information also enabled us to evaluate the phenotypic variation in the context of genetic structure in *L. thermotolerans*, shaped by domestication and allopatric differentiation^4^. Colonisation of a given ecological niche is known to lead to evolutionary differentiation, harnessing adaptation to specific environmental constraints^41^. If such a niche is anthropic, this process can be seen as domestication, either inadvertent or intentional. Signatures of domestication have been confirmed at a genetic level for several other yeast species, i.e. *S. cerevisiae*^42–44^, *S. uvarum*^45^ and *T. delbrueckii*^46^. In *S. cerevisiae*, the genetic differentiation of wild and industrial subpopulations (e.g. winemaking, brewing, baking) was found to be largely reflected at the phenotypic level, with industry-specific selection for stress tolerance, sugar consumption and flavour production^44^. Research has, moreover, highlighted the ‘degrees’ of *S. cerevisiae* domestication; it is the strongest in beer strains, which showed niche specialisation, i.e. decreased ability to grow in nature-like environments as a result of continuous cultivation in mild conditions related to brewing^44^. In contrast, wine strains displayed superior performance across a range of stressors, encountered both in winemaking (e.g. osmotic and ethanol stress) and in nature, likely reflective of the seasonality of winemaking practice^44^. Our previous plate-based phenotyping of *L. thermotolerans* strains using different carbon sources and physicochemical conditions revealed an overall prolific growth of ‘domestic’ groups (harbouring mainly oenological isolates) that might have contributed to their intra-continental dispersal^4^, and in the current study all strains were capable of proliferating in the oenological environment, altogether suggesting an absence of niche specialisation. Nevertheless, the distinct phenotypic performance of *L. thermotolerans* genetic groups, driven by strain fermentation performance and production of (non-)volatile metabolites, was apparent. Notably, two major genetic groups mainly comprised of natural isolates, ‘Americas’ and ‘Canada trees’, showed an overall inferior fermentation performance compared to the ‘domestic’ and ‘mixed’ groups, in terms of lower rate/extent of sugar catabolism (Figs 1 and 2). Moreover, the genetic separation of two ‘domestic’ *L. thermotolerans* groups was also evident at a phenotypic level, as their behaviour for many traits was clearly contrasting. For instance, ‘Domestic 1’ group formed the lowest concentration of lactate, and thus resulted in the highest pH of wines, while ‘Domestic 2’ strains showed superior lactate production that induced a pronounced acidification (Fig. 2). This further emphasises the applicability of microsatellite genotyping in selection of fit-for-purpose *L. thermotolerans* starter cultures; a ‘Domestic 1’ genotype will likely represent a suboptimal choice if the target outcome is (wine) fermentation acidification. Similarly, an overproduction of acetaldehyde and ethyl acetate by ‘Americas’ strains (Supplementary Fig. S3) potentially excludes their use in wine industry, as the increased concentrations of these compounds are detrimental for wine quality^18^. Together, these observations provide further support for the occurrence of domestication events and geographic differentiation in *L. thermotolerans* population.

In conclusion, this study delivers extensive oenological phenomes of 94 previously genotyped *L. thermotolerans* strains, compared for their overall fermentation performance, production of primary and secondary metabolites and modulations in acidity. As such, it not only paints a comprehensive landscape of intra-specific diversity in *L. thermotolerans*, but also highlights the phenotypic manifestations of the genetic differentiation within this remarkable yeast species.

## Materials and Methods

### Culture conditions and media

The cryo-cultures (−80 °C in 25% glycerol) of 94 *L. thermotolerans* strains (Supplementary Table S1) with pre-determined microsatellite genotypes^4^ were grown on YPD plates (1% yeast extract, 2% peptone, 2% glucose and 2% agar) for 3 days at 24 °C. To establish the inoculation cultures, approximately 10^7^ cells were incubated in 900 μL of YPD in each 2 mL well of a 96-well plate agitated on a rotary shaker. After 24 h incubation at 24 °C, cell densities were determined by flow cytometry (Guava easyCyte 12HT, Merck, NJ, USA) to achieve the final inoculation rates of 10^6^ cell/mL. The filter-sterilised (0.2 uM) Chardonnay juice was sourced from the Coombe vineyard (Waite Campus, The University of Adelaide, SA). The concentrations of sugars (glucose and fructose; ~180 g/L) and yeast assimilable nitrogen (~160 mg/L) in the juice were increased to 236.4 g/L (equimolar amounts of glucose and fructose) and 300 mg/L using glucose and fructose and diammonium phosphate, respectively. The juice had a pH of 3.5 and contained 3.8 g/L of malic acid.

### Fermentation trial set-up and monitoring

A custom-made fermentation platform ‘Tee-bot v.2.0’, built on EVO Freedom workdeck (Tecan, Männedorf, Switzerland), was used to conduct the fermentation trials. The platform allowed for up to 384 fermentations to be simultaneously conducted with automatic sampling at user-defined intervals. Each fermenter (50 mL) contained a magnetic flea and an airlock with a silicon (sampling) septum on top and was fitted into a custom-made sealed rack forming 96-fermenter blocks. The fermenters were aseptically supplemented with 25 mL of Chardonnay juice and inoculated with pre-established cultures so that each 96-fermenter block contained one biological replicate of *L. thermotolerans* strains, with a row-wise randomisation between the blocks (Supplementary Fig. S2). The approximate liquid to headspace ratio was 3:1. Upon inoculation, the triplicate fermentations were incubated at 24 °C under anaerobic conditions self-induced upon depletion of the initial oxygen content. The otherwise static fermentations were magnetically stirred during sampling (300 rpm for 2.5 h) so as to ensure yeast cell resuspension. The aliquots (200 µL) were automatically taken at regular intervals (12 or 24 h) into 300 µL 96-well plates to monitor fermentation progress via microbial growth and total sugar consumption. Fermentations were deemed arrested when sugar concentrations did not decline for two consecutive sampling time-points. The final sample was centrifuged (10 min; 3200 × g) in 50 mL tubes and the supernatant decanted into 10 mL tubes and stored at 4 °C until further analysis.

### Analytical techniques

Upon sampling, microbial growth was estimated at 600 nm (OD_600_) upon 30 s resuspension in a plate reader (Infinite 200 PRO, Tecan, Männedorf, Switzerland). The plates were then centrifuged (3 min; 3200 × g) and appropriately diluted for enzymatic determination of total sugar (glucose + fructose) consumption (K-FRUGL kit, Megazyme, Ireland). The pH of wines was measured with a CyberScan 1100 pH meter (Eutech instruments, Thermo Fischer Scientific, MA, USA) and glucose, fructose, ethanol, glycerol, lactic acid, malic acid and acetic acid were analysed by High Performance Liquid Chromatography (HPLC) using a modified method by Frayne^47^. The Agilent 1100 instrument (Agilent Technologies, Santa Clara, CA, USA) was fitted with a HPX-87H column (300 mm × 7.8 mm; BioRad, Hercules, CA, USA) and a 96-well plate sampler (G1367A). Before injection (20 µL), samples (300 µL) were centrifuged (10 min; 1500 × g) using 0.2 µm 96-well plate filter plates (Acroprep^TM^ Advance, Pall Corporation, NY, USA). The eluent was 2.5 mM H_2_SO_4_, with a 0.5 mL/min flow rate at 60 °C for a 35 min run time. Signals were detected using Agilent G1315B diode array detector (organic acids) and G1362A refractive index detector (hexoses and alcohols). Analytes were quantified using the external calibration curves (R^2^ > 0.99) in ChemStation software (version B.01.03). The determined concentrations of metabolites were used to derive the following parameters: consumed sugar (g/L); yields (g/g or mg/g) of ethanol, glycerol, acetate and lactate, which were calculated from their respective concentrations (g/L or mg/L) and sugar consumption extent (g/L); fermentation purity (FP) was expressed as a ratio of acetic acid (g/L) and ethanol (% v/v) and the extent of fructophilicity (G/F) as a ratio of residual glucose and fructose. Concentrations of pyruvic acid in final wines were measured enzymatically (K-PYRUV kit) using a ChemWell 2910 Autoanalyser (Megazyme, Ireland). Solid phase microextraction – gas chromatography - mass spectrometry (SPME-GC-MS) was used to analyse the volatile composition of the wines. Aliquots of the wines (5 mL) were analysed in a 1:2 dilution with deionised H_2_O, with 3 g NaCl added to each SPME vial (20 mL) prior to sample addition. The samples were spiked with 10 µL of a methanolic mixture of five internal standards at the specified concentrations: d13-hexanol (920 mg/L; CDN Isotopes Inc., Pointe-Claire, Canada); d11-hexanoic acid (930 mg/L; CDN Isotopes Inc.); d16-octanal (82.1 mg/L; CDN Isotopes Inc.); d9-ethyl nonanoate^48^ (9.2 mg/L); d3-linalool (1.73 mg/L; CDN Isotopes Inc.). SPME-GC-MS was carried out using an Agilent 7890 A gas chromatograph equipped with a Gerstel (Mülheim an der Ruhr, Germany) MPS2 auto-sampler and using an Agilent 5975 C mass spectrometer for peak detection and compound identification. The auto-sampler was operated in SPME mode utilizing a 2 cm, 23-Gauge, divinylbenzene-carboxen-polydimethylsiloxane fiber (50/30 μm DVB-CAR-PDMS; Supelco, Bellefonte, PA) for extraction. Volatile compounds were extracted using agitation (250 rpm) at 40 °C for 30 mins. Chromatography was performed using a ZB-Wax column (Phenomenex, NSW, Australia) of length 30 m, internal diameter 0.25 mm and film thickness 0.25 μm using helium (Ultrahigh Purity; Air Liquide, SA, Australia) as a carrier gas at 1.2 mL/min with constant flow. Volatiles were desorbed from the fibre in the GC-inlet (220 °C) for 1 min and separated using the following temperature program: 35 °C for 1.5 min, increasing at 7 °C/min to 245 °C, held isothermally at 245 °C for 3.5 min. The temperature of the transfer line connecting the GC and MS was held at 250 °C. Positive-ion electron impact spectra (70 eV) were recorded in scan mode (range: m/z 35–350, scan rate: 4.45 scans/s).

### GC-MS data processing

The GC-MS data was subjected to multivariate curve resolution alternating least squares (MCR-ALS) analysis according to Schmidtke *et al*.^36^ in MATLAB R2017b (Mathworks, Natic, MA, USA). The total ion chromatograms were manually inspected prior to alignment, resulting in 50 time windows. The pre-processing of chromatograms included smoothing and elimination of contamination ions prior to deconvolution. The 90 features (peaks) retained for further analysis were integrated, and their areas were normalised to the geometric mean of the internal standard peak areas. An offset of 1 was applied to each feature peak area prior to logarithmic transformation (base10), mean centring and Pareto scaling were then applied to the block-scaled peak area matrix to obtain the format used for the statistical analysis. The features’ mass spectra were exported in a format compatible with the National Institute of Standards and Technology (NIST) Mass Spectral Search Program (demo version). The identification was conducted by matching the mass spectra with the NIST-11 Library, resulting in either confirmed identity (CI), tentative identity (TI) or no identity (NI) of the target compounds. The criteria for TI were the mass spectra match scores ≥750 and corresponding Kovats’ retention indices (RI), and for CI the same criteria as for TI alongside a comparison with pure compounds. Tentative and confirmed identification was obtained for 15 and 31 compounds, respectively. The identification criteria were not met (NI) for the remaining 44 compounds, denoted as ‘unknown’.

### Data analysis

Data was analysed with custom-made scripts in R^49^. The microbial growth data (OD_600_ readings) were fitted into a logistic model as per Albertin *et al*.^50^, allowing for the extraction of four population dynamics parameters: lag phase duration (lag OD, h), the maximum growth rate (r OD, maximum number of division/h), the maximum growth (max OD), and the growth time without the lag phase (growth time, h). The sugar consumption data was fitted using a Local Polynomial Regression (*loess* function) to estimate the time required for consumption of 5% (lag AF, h), 30% without the lag AF (T30, h) and 50% without the lag AF (T50, h) of initial sugars, and maximum sugar consumption rate (max flux; maximum g/L sugar consumed per h). Growth and sugar consumption parameters were used to identify outliers (*outlier* function; package psych). Outliers for growth encompassed triplicates of 51–160 and YJS4206, two replicates of YJS4246, and one replicate of each MS91Z4 and YJS4295. For sugar consumption, outliers were triplicates of 51–160 and Y72_132, two replicates of YJS4206, and one replicate of YJS4219. The unsupervised learning analysis self-organising map (SOM) was used for dimensionality reduction of both sugar consumption and growth kinetics (*som* function, som package^51^). SOM was performed on mean kinetics per strain upon excluding the outliers, as they may bias the mapping. Several combinations of x-dimension (1–4) and y-dimension (1–4) of the maps were performed. For each combination, a chi² test was performed to determine whether the corresponding SOM allowed for the discrimination of strains’ genetic groups. For growth kinetics, the lowest p-value (0.19) was obtained for four clusters (x = 2, y = 2). For sugar consumption kinetics, the lowest p-value (3.10e-5) corresponded to a 4-cluster map (x = 1, y = 4).

The variation in each measured and derived parameter (114 parameters) was tested following two factors: strain factor and genetic group factor as determined in Hranilovic *et al*.^4^. For each parameter, the factor effect was tested by either ANOVA (to estimate the percentage of variation explained by each factor) or Kruskal-Wallis (KW) to determine the significance groups (R package agricolae). For both factors and both analyses, the p-values were corrected for multiple tests (*p.adjust* function, Benjamini & Hochberg correction). LDA was performed using *lda* function (R package MASS). Since collinear variables may blur the analysis, combined variables (e.g. G/F, yields) were excluded and the data matrix for LDA thus contained 282 rows (experiments) and 107 variables. A classification algorithm was used (random forest implemented on *cforest* function, R package party) to identify which variables accounted the most for genetic group discrimination, and the importance of each variable was computed using the *varimp* function (R package party). Multiple linear regression analysis was performed to examine the relationships between parameters of interest and the remaining variables. An initial model was performed (*lm* function) containing all explaining variables, followed by a stepwise algorithm (*step* function, mode in both direction), which was used to choose a model based on the Akaike Information Criterion (AIC). Correlations between metabolites of interest were assessed using Spearman’s test.

## Electronic supplementary material

Supplementary Information

## Data Availability

The datasets generated and analysed during the current study are available from the corresponding and leading authors on a reasonable request.
