# Supplementary material for: Oenological traits of Lachancea thermotolerans show signs of domestication and allopatric differentiation
Source: Sci Rep. 2018 Oct 4;8:14812. doi: 10.1038/s41598-018-33105-7 (PMC6172252; doi:10.1038/s41598-018-33105-7)
Supplement: Supplementary file 1 — Supplementary Information [file 41598_2018_33105_MOESM1_ESM.pdf]

Supplementary Information

Oenological traits of *Lachancea thermotolerans* show signs of domestication and allopatric differentiation

Ana Hranilovic[1,2], Joanna M. Gambetta[3], Leigh Schmidtke[1,3], Paul K. Boss[4], Paul R. Grbin[2], Isabelle Masneuf-Pomarede[5,6], Marina Bely[5], Warren Albertin[5,7], Vladimir Jiranek[1,2*]

[1] The Australian Research Council Training Centre for Innovative Wine Production, Adelaide, SA, Australia

[2] Department of Wine and Food Science, The University of Adelaide, Adelaide, SA, Australia

[3] National Wine and Grape Industry Centre, School of Agricultural and Wine Science, Charles Sturt University, Wagga Wagga, NSW, Australia

[4] CSIRO Agriculture and Food, Adelaide, SA, Australia

[5] Unité de recherche Œnologie, Institut de la Science de la Vigne et du Vin, University of Bordeaux, Villenave d'Ornon, France

[6] Bordeaux Sciences Agro, Gradignan, France

[7] ENSCBP, Bordeaux INP, Pessac, France

[*] **Correspondence**

Vladimir Jiranek

vladimir.jiranek@adelaide.edu.au

Supplementary Table 1. List of the studied *L. thermotolernas* strains. Strains of different geographic and isolation niches were divided into nine genetic groups according to Hranilovic et al. (2017).

**Strain**	**Source**	**Isolation location**	**Isolation niche**	**Genetic group**	**References**
11/2-112	University of Debrecen	Slovakia, Mala Trna	grapes, mummified	Mix Europe/N. America	2, 5
11/Z-1	University of Debrecen	Slovakia, Mala Trna	grapes, mummified	Mix Europe/N. America	2, 5
14/1/Z-2	University of Debrecen	Hungary, Erdöbénye	grapes, mummified	Mix E. Europe	2, 5
2/2/Z-8	University of Debrecen	Hungary, Sárazsadány	grapes, mummified	Mix Europe/N. America	2, 5
5/1/Z-7	University of Debrecen	Hungary, Vinicky	grapes, mummified	Mix Europe/N. America	2, 5
50-15	Phaff YCC	USA, California	*Drosophila pseudoobscura*	Americas	2
51-160	Phaff YCC	USA, California	*Drosophila azteca*	Americas	2
51-171	Phaff YCC	USA, California	*Drosophila pseudoobscura*	Hawaii/California	2
60-260	Phaff YCC	USA, California	*Aulacigaster sp.*on *Ulmus carpinifolia* exudate	Americas	2
61-245	Phaff YCC	USA, California	*Aulacigaster sp.*	Americas	2
61-518	Phaff YCC	USA, California	*Drosophila melanogaster*	Americas	2
68-140	Phaff YCC	USA, California	bees	Hawaii/California	2
72-132	Phaff YCC	Hawaii, Ahumoa	*Myoporum sandwicens*exudate	Hawaii/California	2
72-137	Phaff YCC	Hawaii, Ahumoa	*Myoporum sandwicens*exudate	Hawaii/California	2
8/2/Z-3	University of Debrecen	Slovakia, Černochov	grapes, mummified	Mix Europe/N. America	2, 5
8/Z-1	University of Debrecen	Slovakia, Černochov	grapes, mummified	Mix E. Europe	2, 5
9/1/Z-4	University of Debrecen	Slovakia, Mala Trna	grapes, mummified	Mix E. Europe	2, 5
AWRI 2009	AWMCC	Australia, South Australia	grapes	Domestic 1	2
CBS 10516	CBS-KNAW	Ukraine	*Quercus sp.* exudate	Europe oak/France grapes	2

CBS 10517	CBS-KNAW	Ukraine	*Quercus sp.* exudate	Other	2
CBS 10518	CBS-KNAW	Ukraine	*Quercus sp.* exudate	Mix E. Europe	2
CBS 137	CBS-KNAW	Netherlands	date	Domestic 1	1, 2
CBS 2860	CBS-KNAW	Italy, Sardinia	grape must	Domestic 1	1, 2
CBS 2907	CBS-KNAW	South Africa	soil	Domestic 2	1, 2
CBS 5464	CBS-KNAW	Australia	cotton seed	Hawaii/California	2
CBS 6292	CBS-KNAW	Australia	na	Europe oak/France grapes	2
CBS 6340T	CBS-KNAW	Russia	mirabelle plum conserve	Domestic 1	1, 2, 3
CBS 6467	CBS-KNAW	Japan	tree exudate	Other	2
CBS 7772	CBS-KNAW	Brazil	*Uca sp.*	Americas	1, 2
CL 41	University of Leon	Spain	grapes	Domestic 2	2
CL 43	University of Leon	Spain	grapes	Hawaii/California	2
CONCERTO™	CHR Hansen	'Mediterranean country'	na	Domestic 1	2
CRBO L0672	CRBOeno	France, Bordeaux	grapes, fermentation	Mix Europe/N. America	2
DBVPG 10092	DBVPG	Algeria	soil, apple orchard	Domestic 2	2
DBVPG 2551	DBVPG	Italy, Piemonte	wine cv. Barbera	Domestic 2	1, 2
DBVPG 2700	DBVPG	Spain, La Mancha	grapes cv. Airen	Mix Europe/N. America	1, 2
DBVPG 3464	DBVPG	Spain, La Mancha	grapes	Domestic 1	1, 2
DBVPG 3466	DBVPG	Spain, La Mancha	grapes	Domestic 1	1, 2
DBVPG 3469	DBVPG	Spain, La Mancha	grapes	Mix Europe/N. America	1, 2
DBVPG 4035	DBVPG	ex Yugoslavia	grapes, must	Domestic 1	1, 2
DBVPG 6322	DBVPG	Italy	grapes	Domestic 2	2
DBVPG 6326	DBVPG	Italy	grapes, raisins	Domestic 2	2
Fin. 89-2	na	Finland	*Quercus sp.*exudate	Europe oak/France grapes	1, 2, 3
FRI10C.1	NCYC	UK, Fritham	*Quercus sp.*	Europe oak/France grapes	2, 4
HU 2511	BOKU	Austria	grapes	Domestic 2	2

ISVV Ltyq25	ISVV	France, Sauternes	grapes, high sugar must	Europe oak/France grapes	2
ISVV Ltyq3	ISVV	France, Sauternes	grapes, high sugar must	Europe oak/France grapes	2
ISVV Ltyq36	ISVV	France, Sauternes	grapes, high sugar must	Europe oak/France grapes	2
JCB1	ISVV	France, Sauternes	grapes, high sugar must	Domestic 1	2
KEH.34.B.3	na	USA, Missouri	grapes, fermentation	Canada trees	1, 2
LEVULIA® ALCOMENO	AEB	France, Burgundy	grapes, fermentation	Europe oak/France grapes	2
LL12-031	LL	Canada	*Quercus sp.*tree bark	Canada trees	2
LL12-040	LL	Canada	*Acer sp.* bark	Mix Europe/N. America	2
LL12-041	LL	Canada	*Quercus sp.*bark	Mix Europe/N. America	2
LL12-056	LL	Canada	planted *Quercus sp.*bark	Canada trees	2
LL13-038	LL	USA, Massachusetts	*Quercus sp.*bark	Mix Europe/N. America	2
LL13-189	LL	Canada, New-Brunswick	*Quercus sp.*bark	Canada trees	2
LL13-194	LL	Canada, New-Brunswick	*Quercus sp.*bark	Canada trees	2
MB10D.1	NCYC	France, Montbarri	*Quercus sp.*	Europe oak/France grapes	2, 4
MUCL 31341	MUCL	Italy	wine	Domestic 1	1, 2
MUCL 31342	MUCL	Italy	wine	Domestic 1	2
MUCL 47720	MUCL	Italy	wine	Domestic 1	2
NCAIM Y.00775	NCAIM	Hungary, Babat	*Carpinus betulu* exudate	Mix Europe/N. America	2
NCAIM Y.00798	NCAIM	Hungary, Csikóváralja	brown rotten *Quercus sp.*	Mix Europe/N. America	2
NCAIM Y.00873	NCAIM	Hungary, Budapest	rotten material of a cavity of *Betula pendula*	Mix E. Europe	2
NCAIM Y.01703	NCAIM	Hungary, Nagyeged	grapes	Mix Europe/N. America	2
NRLL Y-2193	NRRL/ARS	USA, California	*Drosophila pseudoobscura*	Americas	2
NRLL Y-27329	NRRL/ARS	USA, West Virginia	grapes	Domestic 2	2

NZ156	CRPR	New Zealand	grapes cv. Chardonnay	Domestic 1	2
OCK6C.1	NCYC	UK, Ocknell	*Quercus sp.*	Europe oak/France grapes	2, 4
OSU A	OSU	USA, Oregon	grapes	Mix Europe/N. America	2
PLU5B.1	NCYC	UK, East Sussex	*Quercus sp.*	Europe oak/France grapes	2, 4
PYR14B.1	NCYC	Greece, Pyradikia	*Quercus sp.*	Domestic 1	2, 4
T 13/17 F	University of the Republic	Uruguay	grapes cv. Tannat	Domestic 2	2
TAX9D.1	NCYC	Greece, Taxiarchis	*Quercus sp.*	Mix Europe/N. America	2, 4
UNIFG 16	UNIFG	Italy	wine	Domestic 2	2
UNIFG 17	UNIFG	Italy	wine	Domestic 2	2
UNIFG 18	UNIFG	Italy	wine	Domestic 2	2
UNIFG 22	UNIFG	Italy	wine	Domestic 2	2
UNIFG 26	UNIFG	Italy	wine	Domestic 1	2
UNIFG 28	UNIFG	Italy	wine	Domestic 2	2
UNIFG 32	UNIFG	Italy	wine	Domestic 2	2
UWOPS 79-110	UWOPS	Canada, Ontario	black knot, *Prunus virginiana*	Canada trees	1, 2
UWOPS 79-164	UWOPS	Canada, Ontario	black knot, *Prunus virginiana*	Canada trees	1, 2
UWOPS 79-195	UWOPS	Canada, Ontario	black knot, *Prunus virginiana*	Canada trees	1, 2
UWOPS 83-1097.1	UWOPS	Cayman Islands	black knot, *Prunus virginiana*	Americas	1, 2, 3
UWOPS 83-1101.1	UWOPS	Cayman Islands	*Gitona americana*, *Opuntia stricta*	Americas	1, 2
UWOPS 85-312.1	UWOPS	USA, Arizona	*Drosophila carbonaria*, *Prosopis juliflora*	Americas	1, 2
UWOPS 85-51.1	UWOPS	USA, Florida	*Opuntia cubensis*	Americas	1, 2
UWOPS 90-10.1	UWOPS	Bahamas, Exumas Cays	Columnar cactus	Americas	1, 2
UWOPS 91-910.1	UWOPS	Hawaii, Ahumoa	flux (pink), *Myoporum*	Hawaii/California	1, 2
UWOPS 91-912.1	UWOPS	Hawaii, Ahumoa	flux (white), *Myoporum*	Hawaii/California	1, 2
UWOPS 94-426.2	UWOPS	Mexico, Jalisco	distillery, agave must	Domestic 1	1, 2

Yal. 87-1	na	Russia, Crimea	*Quercus sp.* exudate	Europe oak/France grapes	2, 3

Phaff YCC – Phaff Yeast Culture Collection, University of California, Davis, USA; **AWMCC** – AWRI Wine Microorganism Culture Collection, Australia; **CBS-KNAW** – Centraalbureau voor Schimmelcultures – Koninklijke Nederlandse Akademie van Wetenschappen, Netherlands; **CRBOeno** - Centre de Ressources Biologiques OEnologie, France; **DBVPG** The Industrial Yeasts Collection DBVPG, Italy; **NCYC** – National Collection of Yeast Cultures, UK;, Italy; **BOKU** – Universität für Bodenkultur Wien, Austria; **ISVV** – Institut des Sciences de la Vigne et du Vin, France; **LL** – Landry Lab, Canada; **MUCL** – Mycothèque de l'Université catholique de Louvain, Belgium; **NCAIM** – National Collection of Agricultural and Industrial Microorganisms, Hungary; **NRRL/ARS** – NRRL Agriculture Research Service Culture collection, USA; **CRPR** – Centre de Recherche Pernod-Ricard, France; **OSU** – Oregon State University, USA; **UNIFG –** University of Foggia; **UWOPS** – Culture Collection of the University of Western Ontario; na – not available; [1] Freel, K. C., Friedrich, A., Hou, J. & Schacherer, J. Population genomic analysis reveals highly conserved mitochondrial genomes in the yeast species *Lachancea thermotolerans. Genome Biol. Evol.* **6**, 2586-2594, doi:10.1093/gbe/evu203 (2014); [2] Hranilovic, A., Bely, M., Masneuf-Pomarede, I., Jiranek, V. & Albertin, W. The evolution of *Lachancea thermotolerans* is driven by geographical determination, anthropisation and flux between different ecosystems. *Plos One* **12**, e0184652, doi:10.1371/journal.pone.0184652 (2017); [3] Naumova, E. S., Serpova, E. V. & Naumov, G. I. Molecular systematics of *Lachancea* yeasts. *Biochemistry (Moscow)* **72**, 1356-1362 (2007); [4] Robinson, H. A., Pinharanda, A. & Bensasson, D. Summer temperature can predict the distribution of wild yeast populations. *Ecol. Evol.* **6**, 1236-1250 (2016); [5] Sipiczki, M. Overwintering of vineyard yeasts: Survival of interacting yeast communities in grapes mummified on vines. *Front. Microbiol.* **7**, 212, doi:10.3389/fmicb.2016.00212 (2016).

Supplementary Table 2. Analysed volatile compounds in *L. thermotolerans* wines displaying significant (p-value < 0.05) and non-significant strain effect divided into chemical classes. The tentative identification (TI) was accomplished via corresponding Kovats' retention indices (RI) obtained with an equivalent stationary phase (in italics) and/or mass spectra match scores ≥ 750, and confirmed (confirmed identification; CI) via comparison with pure compounds. The unknown compounds (no identification, NI) are numbered based on the chromatographic elution profile.

No.	Compound	Formula	CAS	Kovats' RI	Identification
Significant strain effect					
	**Alcohols**				
1	butanol	$C_4H_{10}O$	71-36-3	1143	CI
2	isobutanol	$C_4H_{10}O$	78-83-1	1089	CI
3	isoamyl alcohol	$C_5H_{12}O$	123-51-3	1222	CI
4	2-methyl-1-butanol	$C_5H_{12}O$	137-32-6	1219	CI
5	hexanol	$C_6H_{14}O$	111-27-3	1349	CI
6	3-methyl-1-pentanol	$C_6H_{14}O$	589-35-5	*1334*[h]	TI
7	2-ethyl-1-hexanol	$C_8H_{18}O$	104-76-7	*1483*[i]	TI
8	octanol	$C_8H_{18}O$	111-87-5	1560	CI
9	2-phenylethanol	$C_8H_{10}O$	60-12-8	1901	CI
10	4-methyl-benzenemethanol	$C_8H_{10}O$	589-18-4	*1967*[a]	TI
11	nonanol	$C_9H_{20}O$	143-08-8	1647	CI
12	decanol	$C_{10}H_{22}O$	112-30-1	1755	CI
	**Esters**				
13	ethyl acetate	$C_4H_8O_2$	141-78-6	882	CI
14	ethyl propanoate	$C_5H_{10}O_2$	105-37-3	954	CI
15	isobutyl acetate	$C_6H_{12}O_2$	110-19-0	1011	CI
16	isoamyl acetate	$C_7H_{14}O_2$	123-92-2	1111	CI
17	diethyl succinate	$C_8H_{14}O_4$	123-25-1	1670	CI
18	amyl lactate	$C_8H_{16}O_3$	6382-06-5		TI
19	2-phenylethyl acetate	$C_{10}H_{12}O_2$	103-45-7	1802	CI
20	ethyl octanoate	$C_{10}H_{20}O_2$	106-32-1	1414	CI
21	ethyl decanoate	$C_{12}H_{24}O_2$	110-38-3	1629	CI
22	ethyl 9-decenoate	$C_{12}H_{22}O_2$	67233-91-4	*1688*[b]	TI

	**Acids**				
23	4-hydroxy-butanoic acid	$C_4H_8O_3$	591-81-1		TI
24	hexanoic acid	$C_6H_{12}O_2$	142-62-1	1860	CI
25	octanoic acid	$C_8H_{16}O_2$	124-07-2	2076	CI
26	decanoic acid	$C_{10}H_{20}O_2$	334-48-5	2295	CI
27	dodecanoic acid	$C_{12}H_{24}O_2$	143-07-7	2488	CI
	**Aromatic hydrocarbons**				
28	1,2,4-trimethyl-benzene	C_9H_{12}	95-63-6	*1277*[c]	TI
29	1-ethyl-2,4-dimethyl benzene	$C_{10}H_{14}$	874-41-9	*1348*[d]	TI
30	1,3-bis(1,1-dimethylethyl)-benzene	$C_{14}H_{22}$	1014-60-4		TI
	**Aldehydes**				
31	acetaldehyde	C_2H_4O	75-07-0	*744*[e]	TI
32	4-methyl-benzaldehyde	C_8H_8O	104-87-0	*1656*[f]	TI
	**Norisoprenoid**				
33	β-damascenone	$C_{13}H_{18}O$	23726-93-4	1830	CI
	**Terpenols**				
34	β-citronellol	$C_{10}H_{20}O$	106-22-9	1779	CI
	**Ketone**				
35	4-methyl-2-heptanone	$C_8H_{16}O$	6137-06-0	*1206*[g]	TI
	**Unknowns**				
36-58	F8, F10, F19, F22, F24, F27, F30, F40, F43, F45, F46, F47, F48, F50, F53, F54, F56, F77, F80, F83, F85, F86, F90				NI
Non-significant strain effect					
	**Alcohols**				

59	propanol	C_3H_8O	71-23-8	1030	CI
60	3-ethoxy-1 propanol	$C_5H_{12}O_2$	111-35-3	1377	CI
61	(Z)-3-hexen-1-ol	$C_6H_{12}O$	928-96-1	1396	CI
	**Esters**				
62	ethyl butanoate	$C_6H_{12}O_2$	105-54-4	1028	CI
63	ethyl hexanoate	$C_8H_{16}O_2$	123-66-0	1592	CI
	**Aromatic hydrocarbons**				
64	1-ethyl-3-methyl-benzene	C_9H_{12}	620-14-4	*1224*[c]	TI
65	2-ethyl-1,4-dimethyl-benzene	$C_{10}H_{14}$	1758-88-9	*1343*[d]	TI
	**Aldehydes**				
66	nonanal	$C_9H_{18}O$	124-19-6	1375	CI
67	2,5-benzaldehyde	$C_9H_{10}O$	5779-94-2		TI
	**Ketone**				
68	2-nonanone	$C_9H_{18}O$	821-55-6	1375	CI
	**Terpenols**				
69	terpineol	$C_{10}H_{18}O$	8006-39-1	1690	CI
	**Unknowns**				
70-90	F5, F13, F17, F20, F26, F31, F32, F41, F42, F52, F55, F64, F65, F66, F72, F73, F74, F79, F81, F84, F87				NI

[a] Umano, K., Nakahara, K., Shoji, A. & Shibamoto, T. Aroma chemicals isolated and identified from leaves of *Aloe arborescens* Mill. Var. *natalensis* Berger. *J. Agric. Food Chem.* **47**, 3702-3705 (1999); [b] Zhao, Y., Xu, Y., Li, J., Fan, W. & Jiang, W. Profile of volatile compounds in 11 brandies by headspace solid-phase microextraction followed by gas chromatography-mass spectrometry. *J. Food Sci.* **74,** 90-99, doi:10.1111/j.1750-3841.2008.01029.x (2009); [c] Toth, T. Use of capillary gas chromatography in collecting retention and chemical information for the analysis of complex petrochemical mixtures. *J. Chromatogr.* A **279**, 157-165 (1983); [d] Umano, K., Hagi, Y., Nakahara, K., Shoji, A. & Shibamoto, T. Volatile chemicals identified in extracts from leaves of Japanese mugwort

(*Artemisia princeps* Pamp.). *J. Agric. Food Chem.* **48**, 3463–3469 (2000); [e] Umano, K., Shoji, A., Hagi, Y. & Shibamoto, T. Volatile constituents of peel of quince fruit, *Cydonia oblonga* Miller. *J. Agric. Food Chem.* **34**, 593–596 (1986); [f] Steullet, P. & Guerin, P. M. Identification of vertebrate volatiles stimulating olfactory receptors on tarsus I of the tick *Amblyomma variegatum* Fabricius (Ixodidae). *J. Comp. Physiol. A* **174**, 27-38 (1994); [g] Canuti, V. et al. Headspace solid-phase microextraction - gas chromatography - mass spectrometry for profiling free volatile compounds in Cabernet Sauvignon grapes and vines. *J. Chromatogr.* A **1216**, 3012-3022 (2009); [h] Gurbuz, O., Rouseff, J. M. & Rouseff, R. L. Comparison of aroma volatiles in commercial Merlot and Cabernet Sauvignon wines using gas chromatography - Olfactometry and gas chromatography - Mass spectrometry. *Journal of Agricultural and Food Chemistry J. Agric. Food Chem.* **54**, 3990-3996 (2006); [i] Karlsson, M. F. et al. Plant odor analysis of potato: responce of guatemalan moth to above- and background potato volatiles. *J. Agric. Food Chem.* **57**, 5903-5909 (2009).

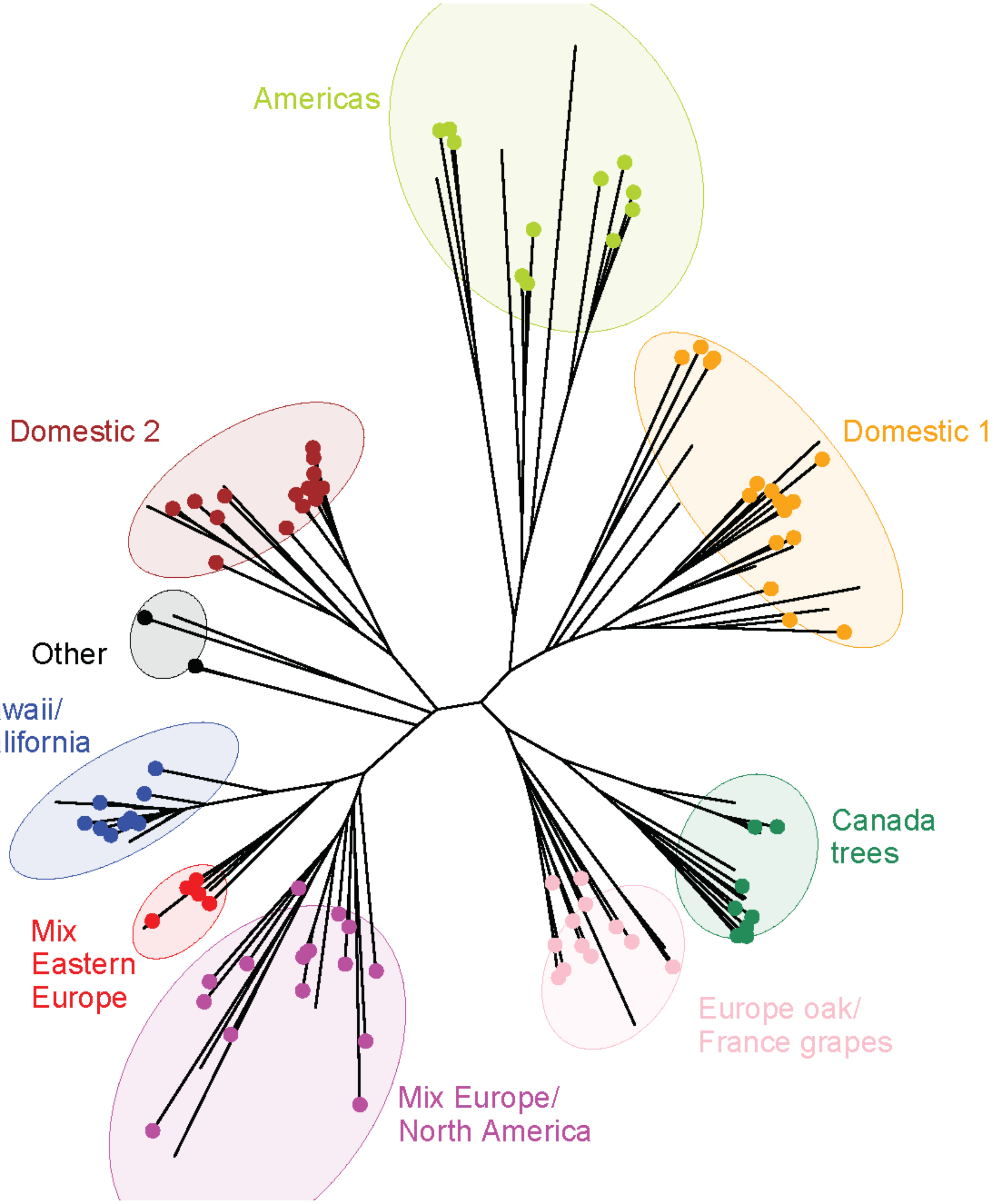

Supplementary Figure 1. Genetic relationships between 94 phenotyped *Lachancea thermotolerans* determined using 14 microsatellite markers.

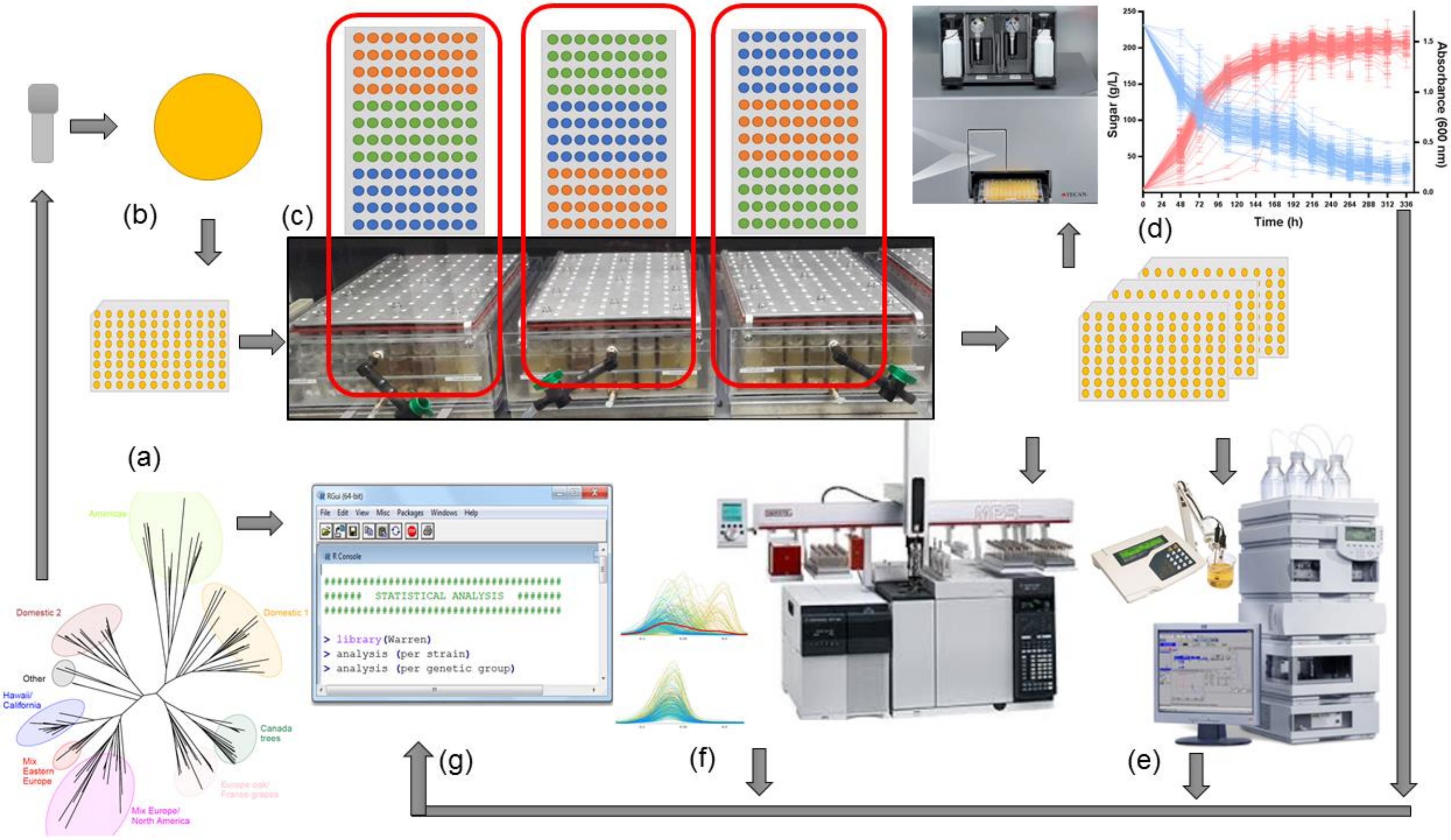

(a)
(b)
(c)
(d)
(e)
(f)
(g)
Americas
Domestic 2
Domestic 1
Other
Hawaii/
California
Canada
trees
Mix
Eastern
Europe
Europe oak/
France grapes
Mix Europe/
North America
Sugar (g/L)
Absorbance (600 nm)
Time (h)
0 24 48 72 96 120 144 168 192 216 240 264 288 312 336
250 200 150 100 50
1.5 1.0 0.5 0.0
RGui (64-bit)
File Edit View Misc Packages Windows Help
R Console
###################################
STATISTICAL ANALYSIS
###################################
> library(Warren)
> analysis (per strain)
> analysis (per genetic group)

Supplementary Figure 2. Schematic representation of the *L. thermotolerans* phenotyping workflow: The cryo-cultures of 94 *L. thermotolerans* strains belonging to nine genetic groups (a) according to Hranilovic et al. (2017) were grown on YPD agar plates and in YPD broth to establish the inoculation cultures for Chardonnay grape juice fermentations (b). The triplicate fermentations were set up in 'Tee-bot v.2.0' using three 96-fermentor blocks (c). Each block contained one biological replicate, with row-wise randomisation between the blocks (indicated with orange, green and blue colour). Such randomisation was maintained for all downstream analysis. Fermentations were monitored regularly via OD_{600} and sugar consumption (d). The final wines were analysed for their pH values, and concentrations of organic acids, hexoses and alcohols via HPLC (e), and volatile composition via SPME-GC-MS (f). All the measured and derived parameters were subjected to appropriate univariate and multivariate statistical analysis (g).

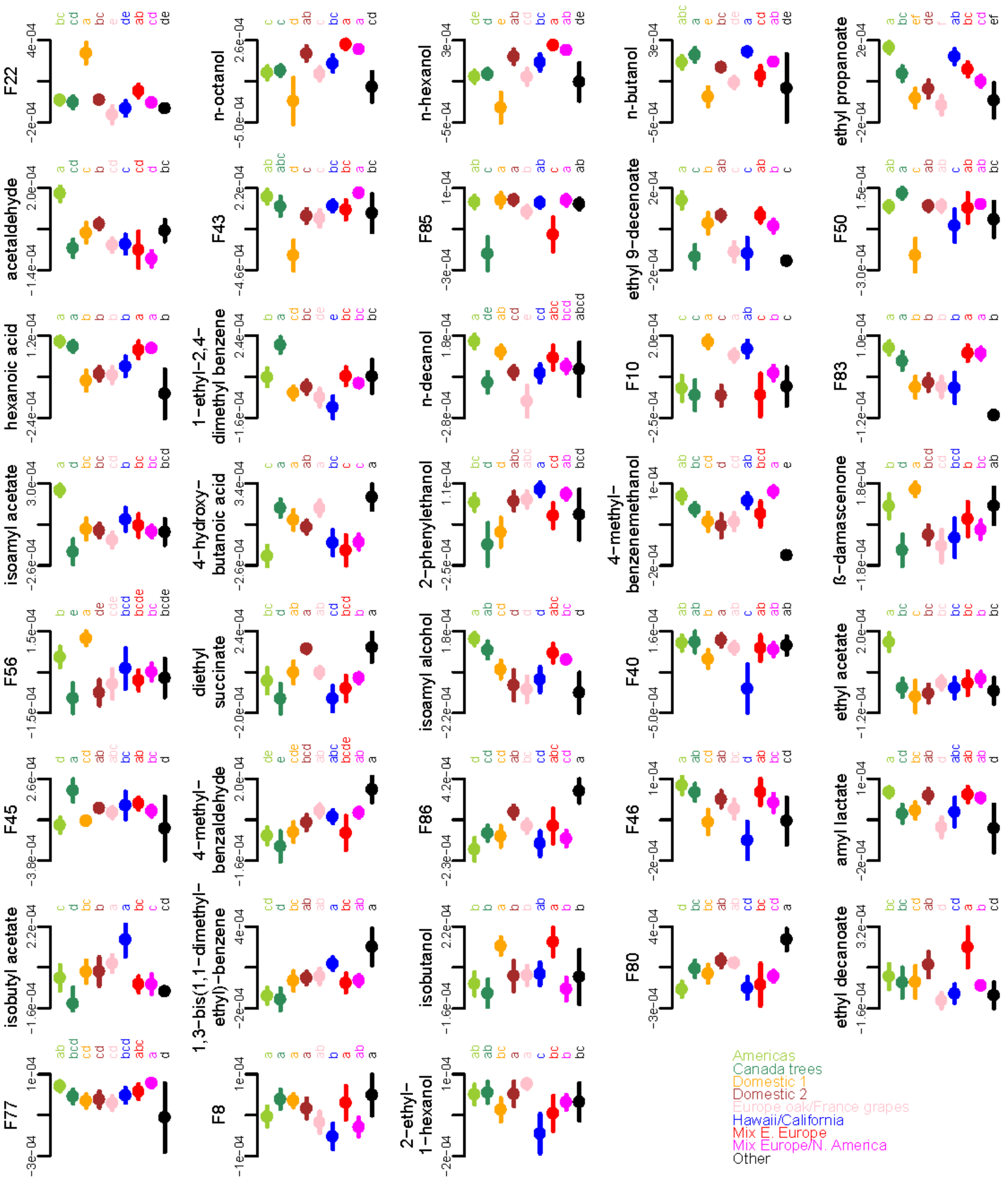

Supplementary Figure 3. The analysed volatile compounds displaying a significant genetic group effect. Dots and bars represent means and standard errors, respectively. Top letters represent significance groups as defined by Kruskal-Wallis test (*agricolae* package, p-value < 0.05 after Benjamini & Hochberg adjustment for multiple comparisons).

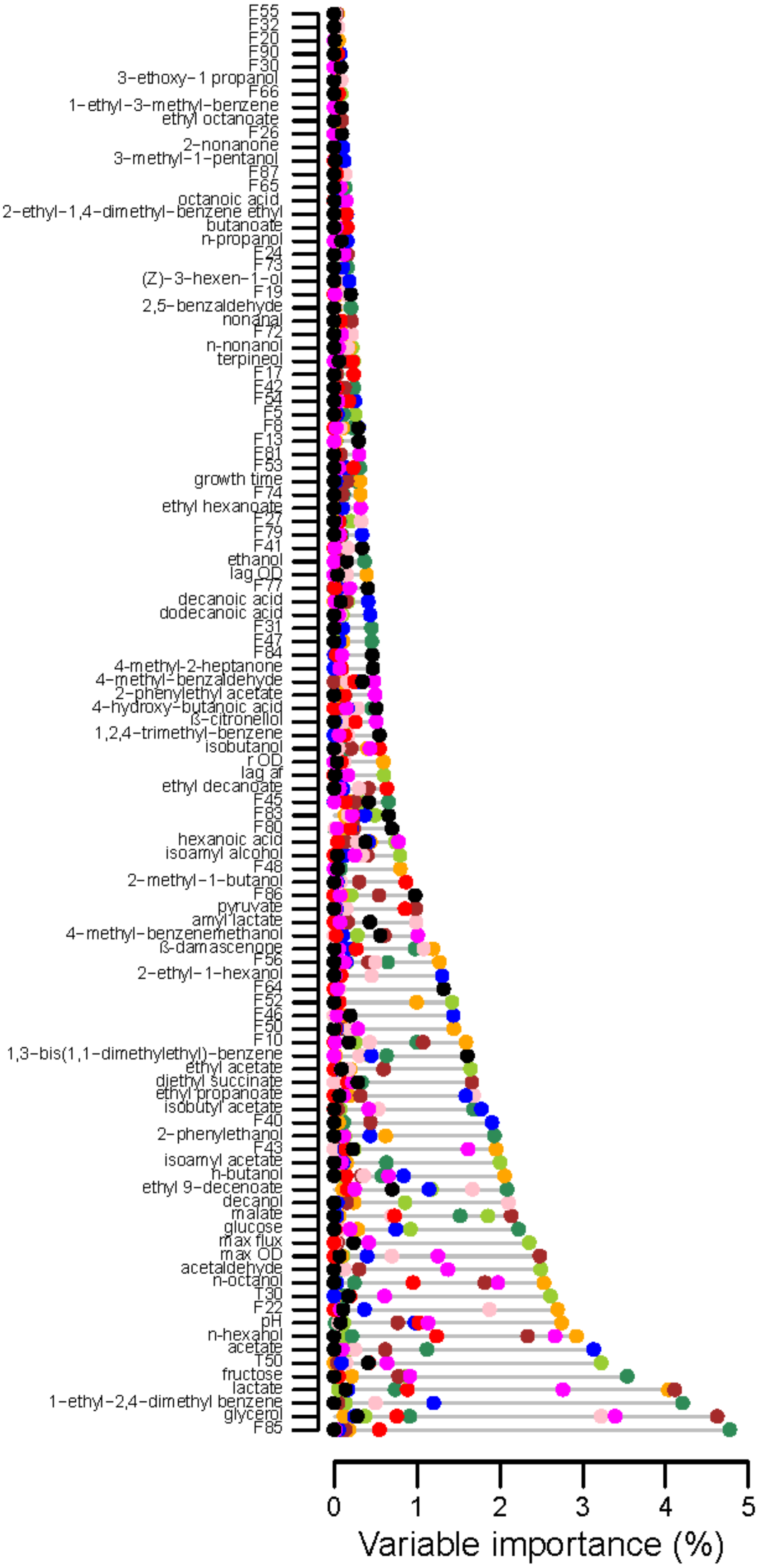

Supplementary Figure 4. The importance of all 107 variable subjected to LDA.

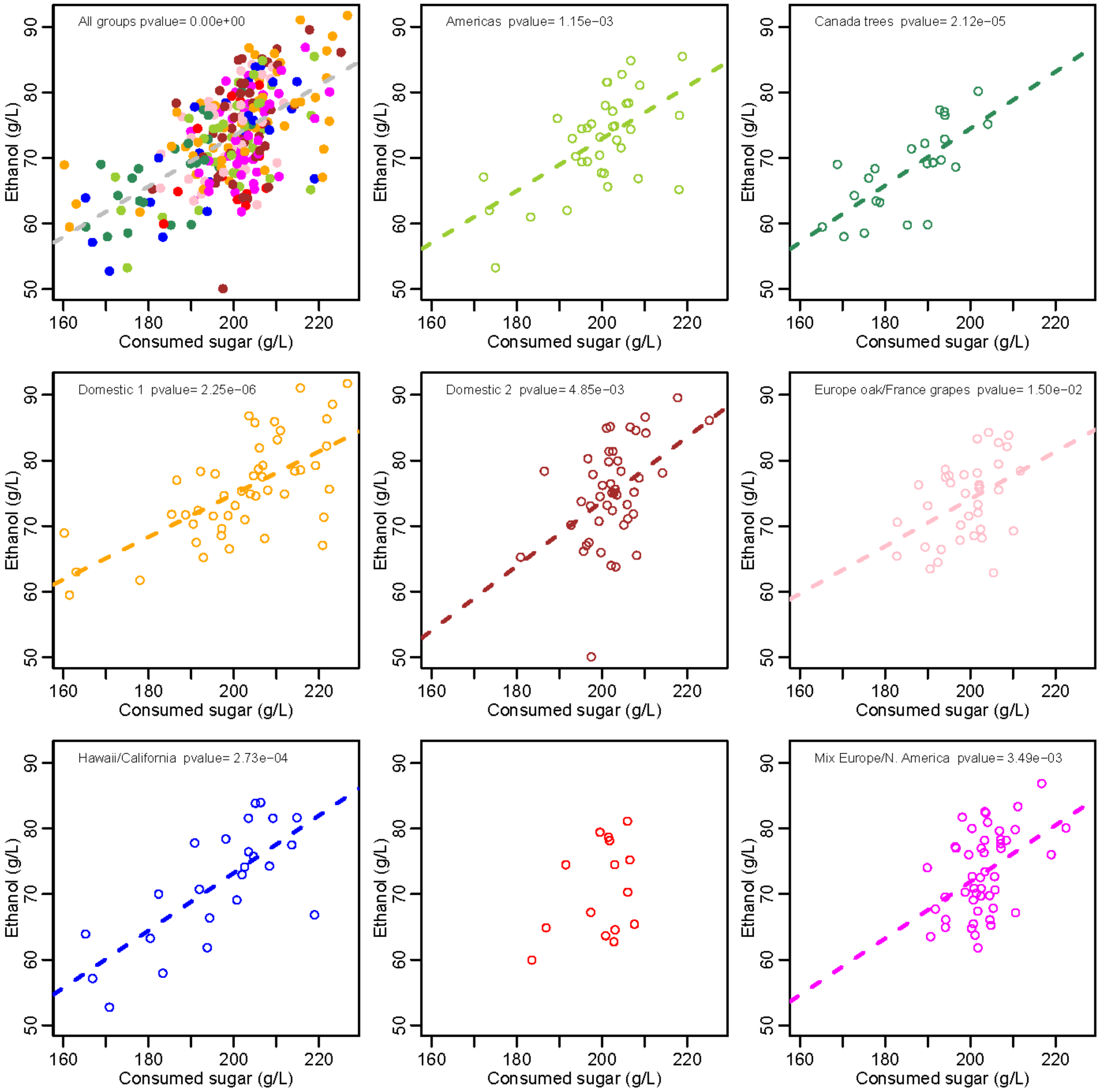

Supplementary Figure 5. Spearman’s correlation test between the selected metabolites: consumed sugar and ethanol.

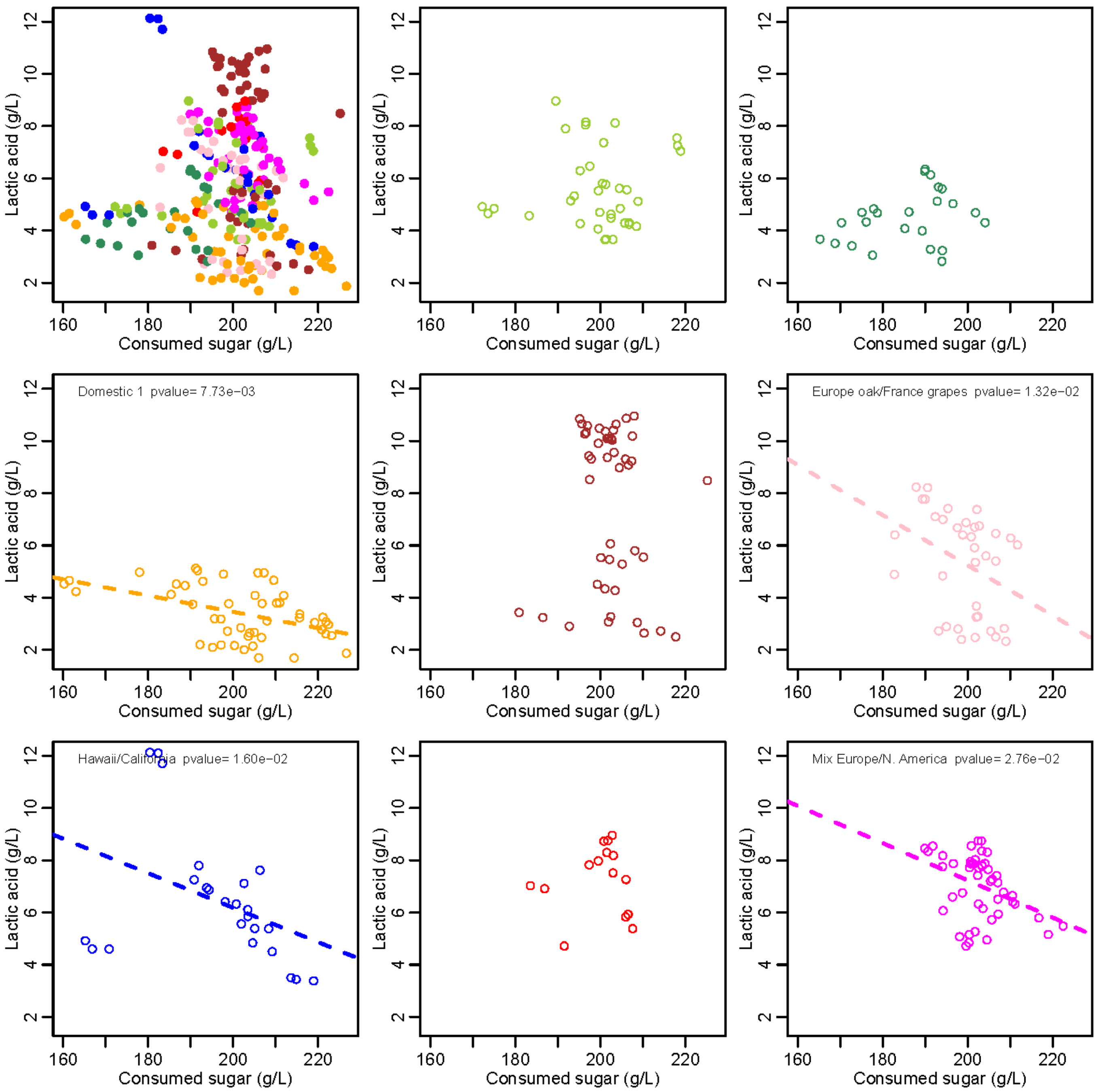

Supplementary Figure 6. Spearman's correlation test between the selected metabolites: consumed sugar and lactic acid.

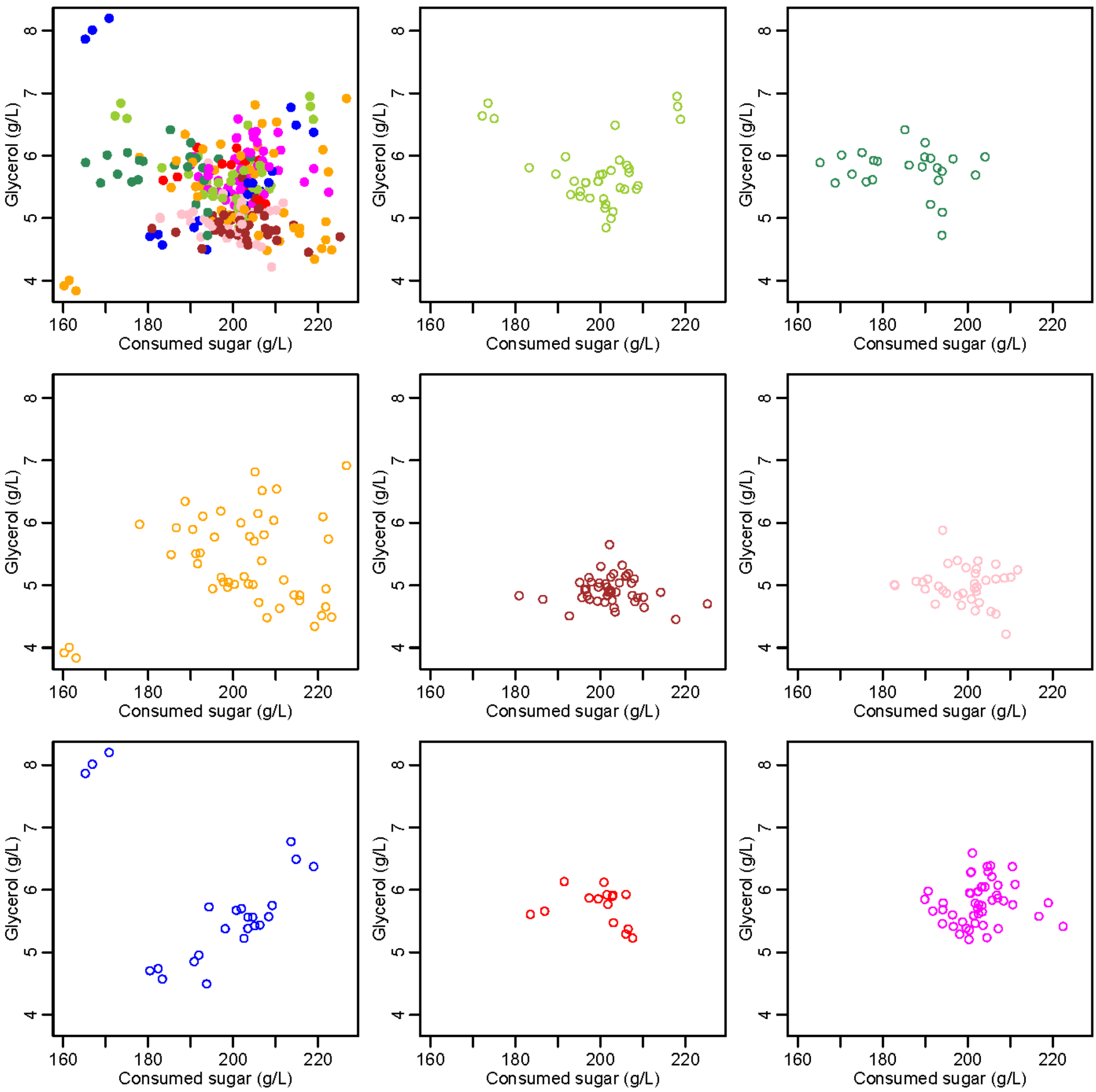

Supplementary Figure 7. Spearman's correlation test between the selected metabolites: consumed sugar and glycerol.

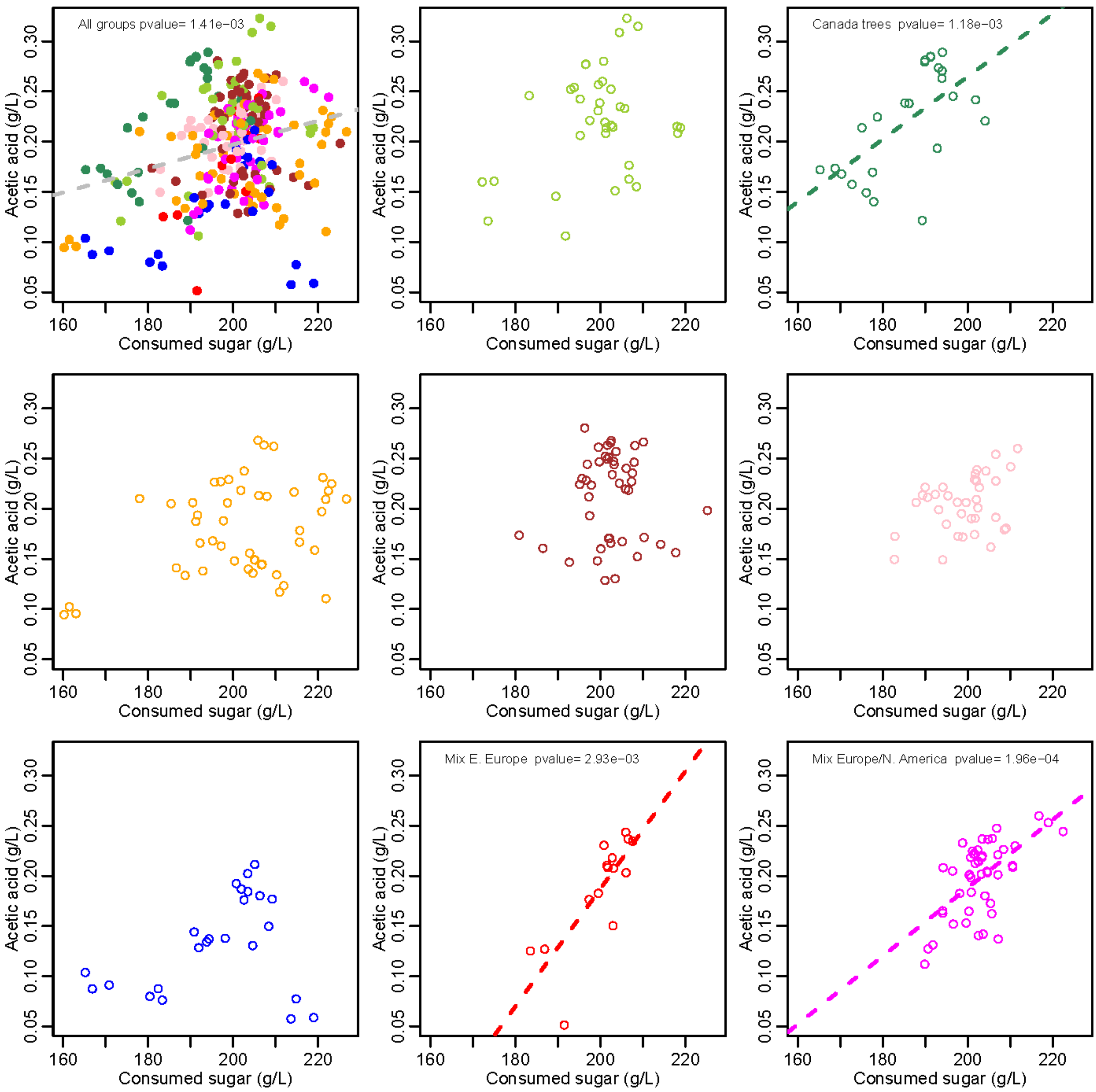

Supplementary Figure 8. Spearman's correlation test between the selected metabolites: consumed sugar and acetic acid.

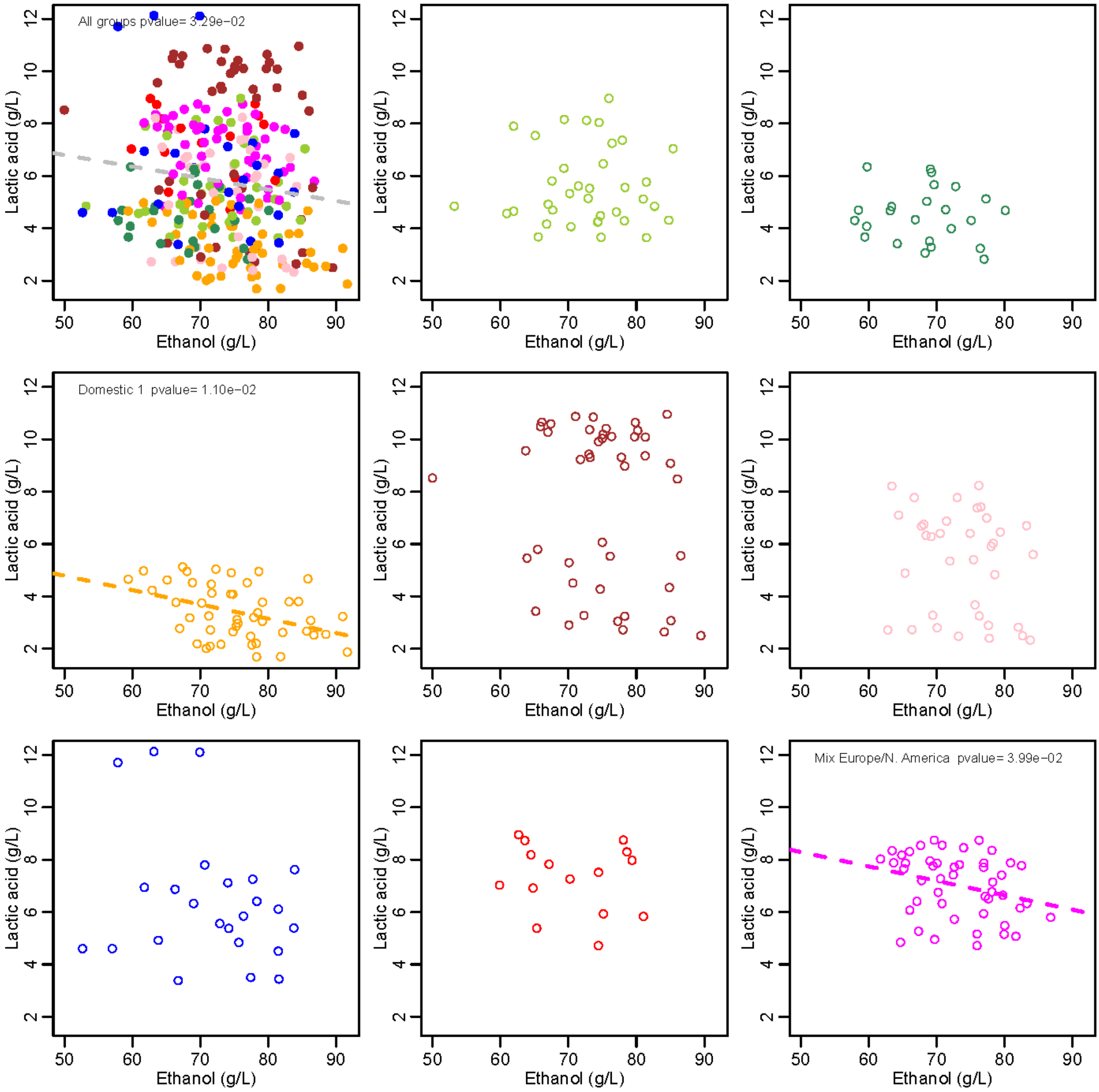

Supplementary Figure 9. Spearman's correlation test between the selected metabolites: ethanol and lactic acid.

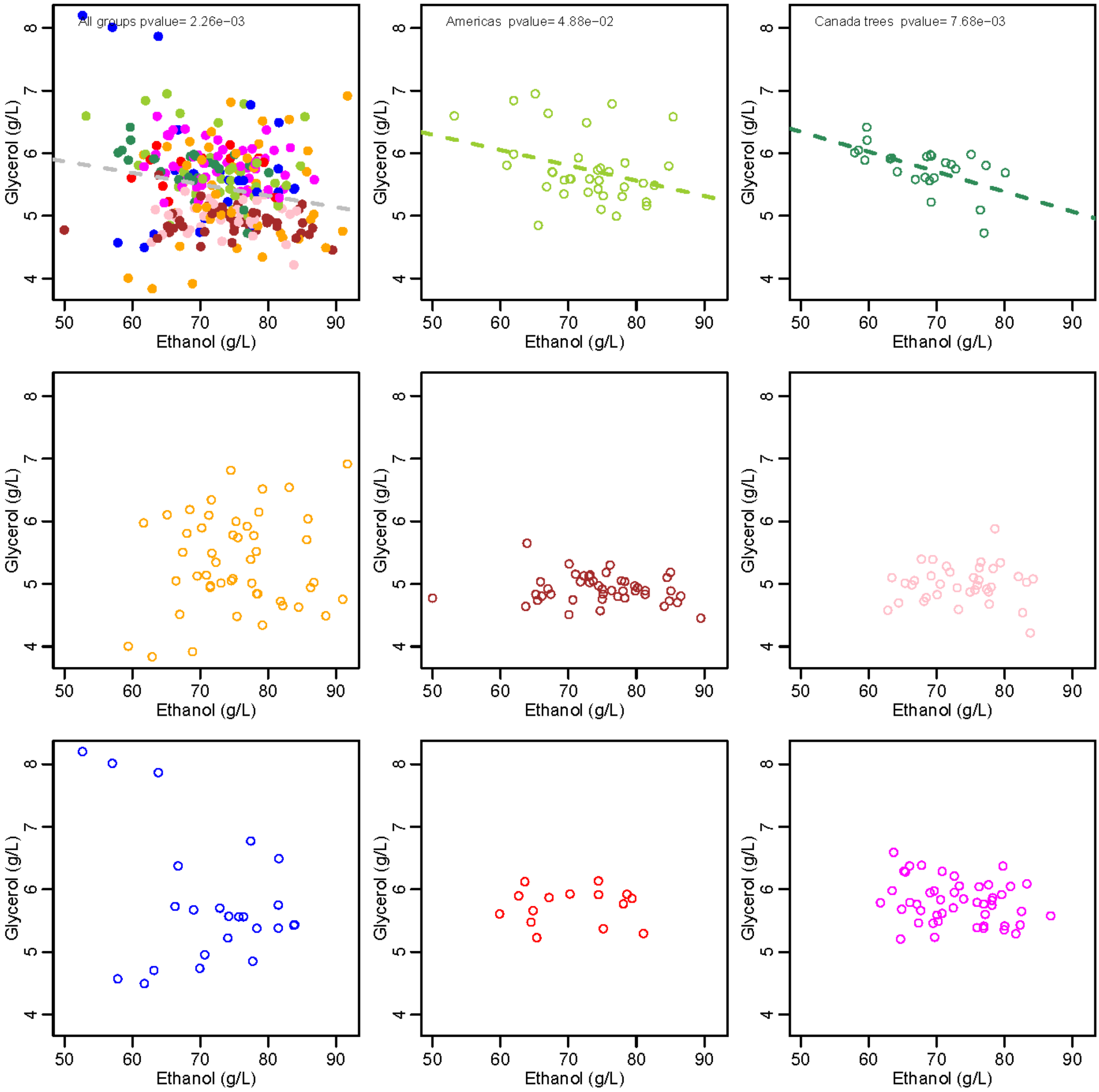

Supplementary Figure 10. Spearman’s correlation test between the selected metabolites: ethanol and glycerol.

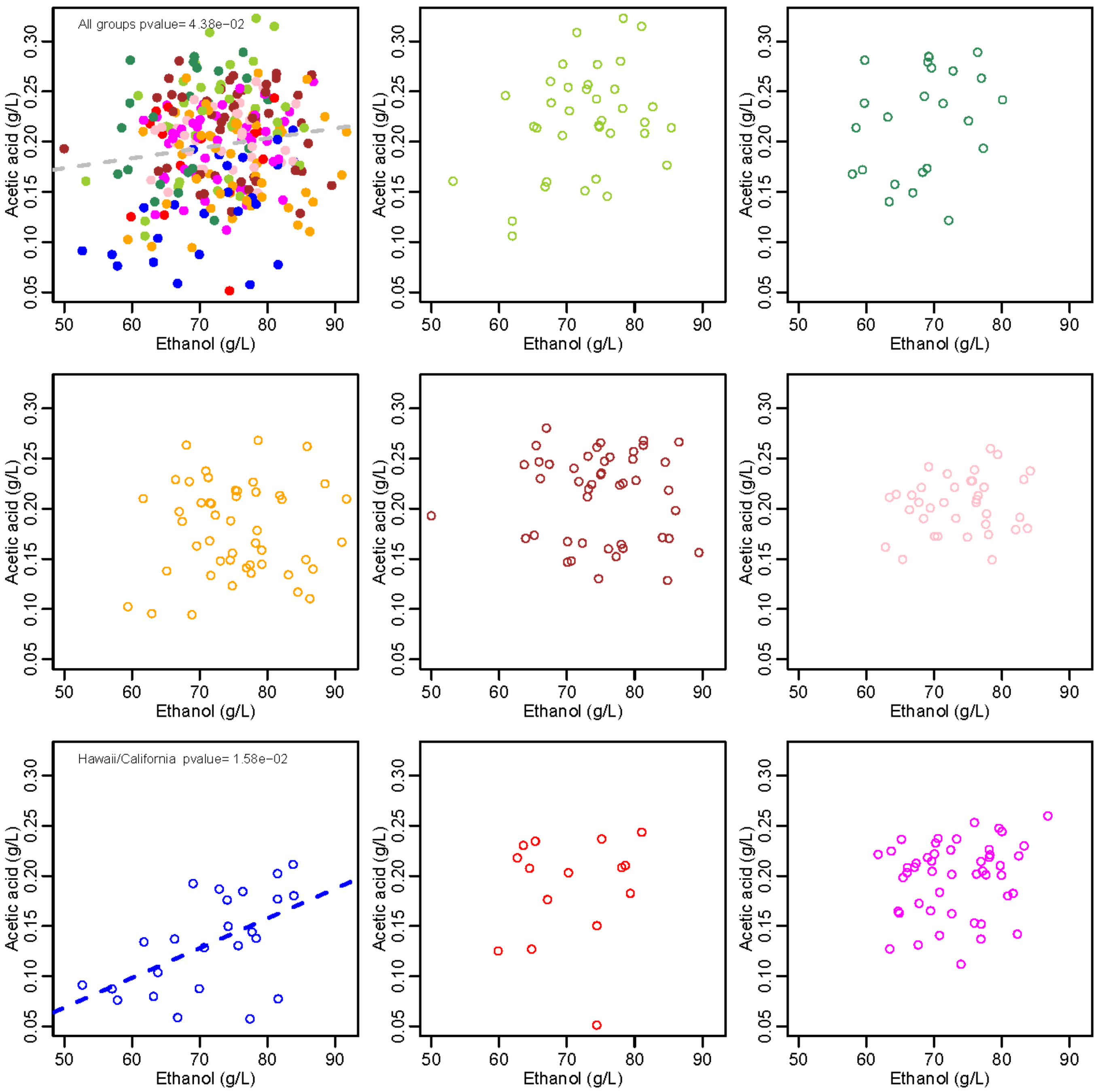

Supplementary Figure 11. Spearman's correlation test between the selected metabolites: ethanol and acetic acid.

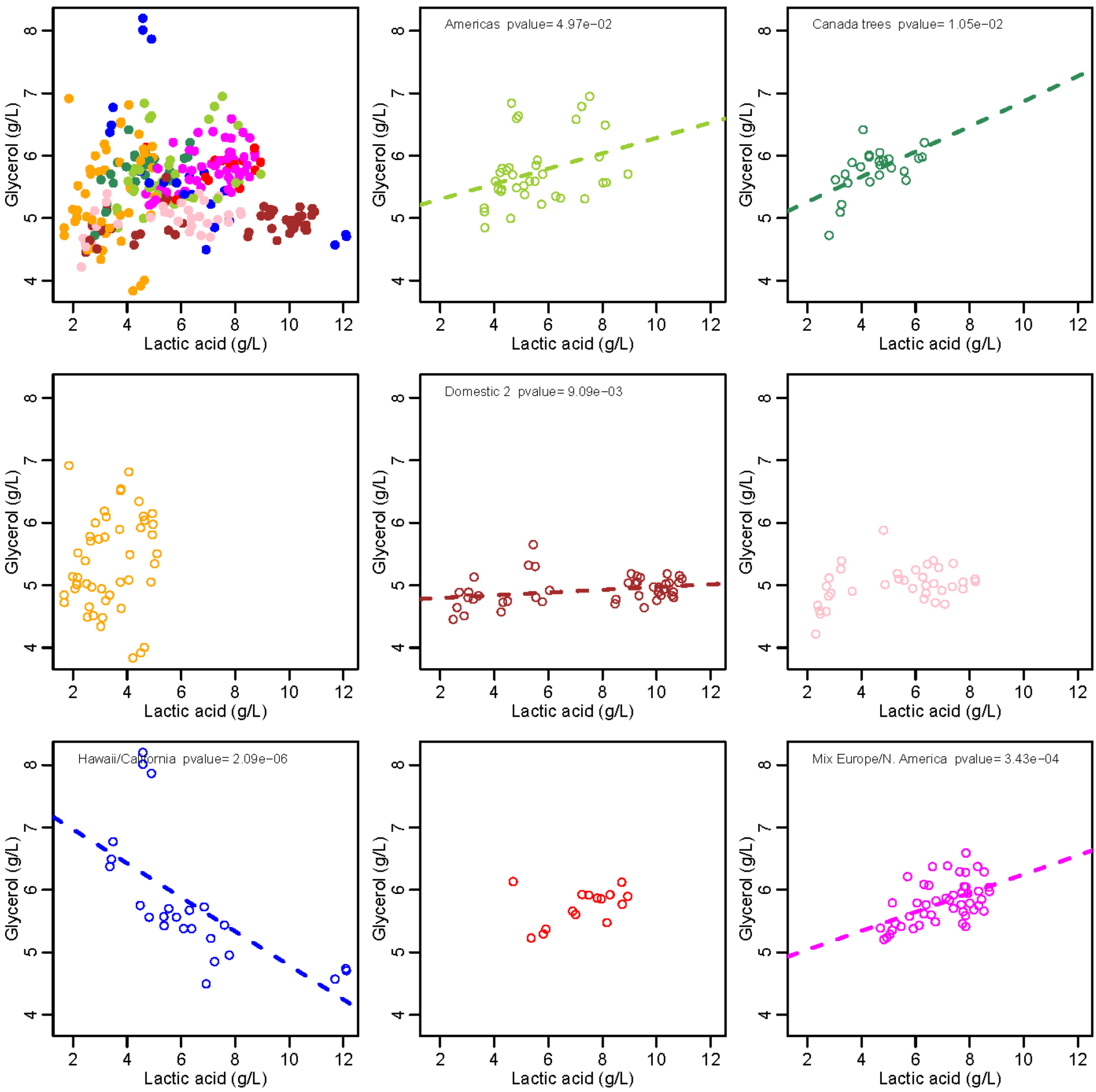

Supplementary Figure 12. Spearman's correlation test between the selected metabolites: lactic acid and glycerol.

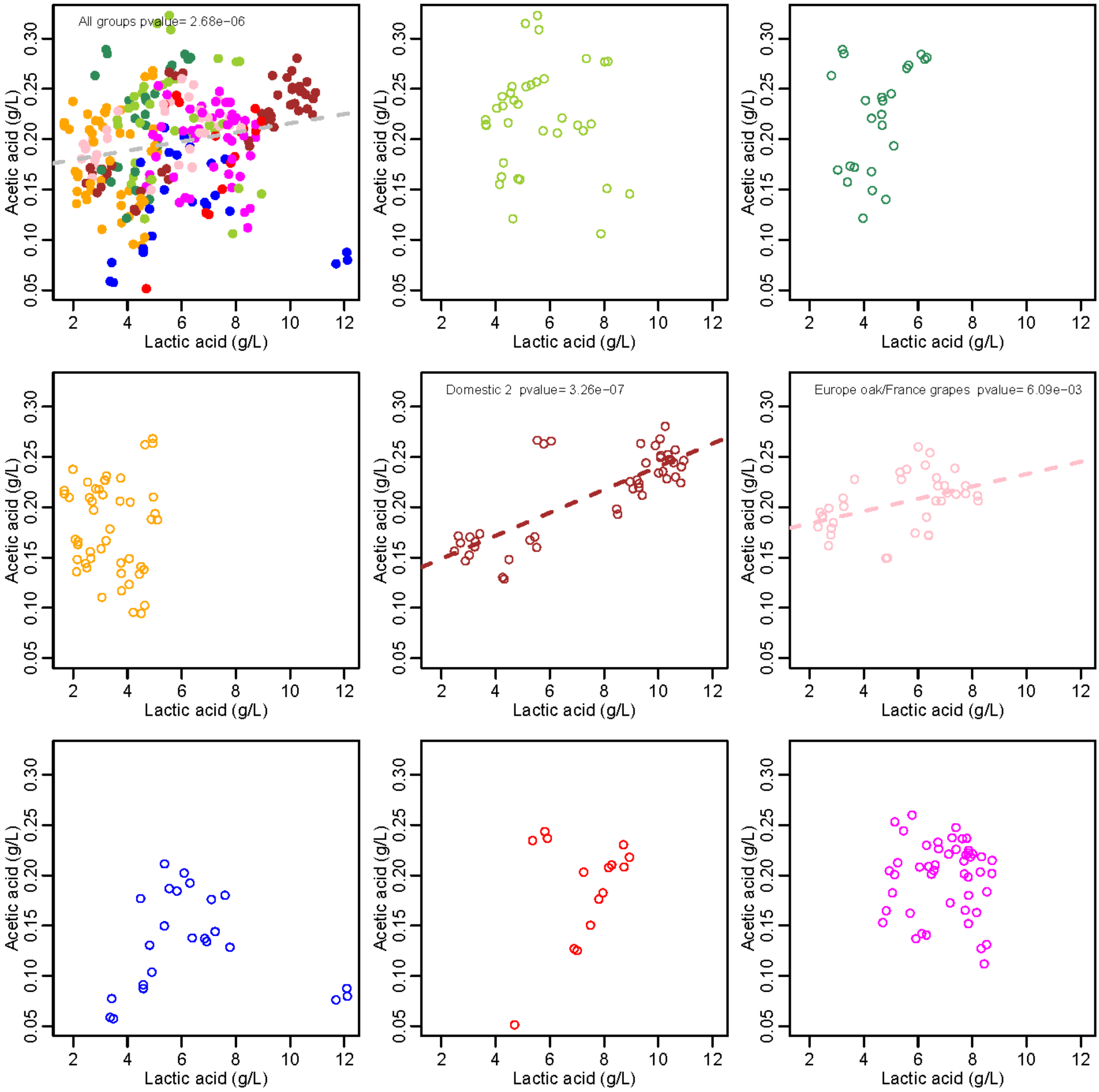

Supplementary Figure 13. Spearman's correlation test between the selected metabolites: lactic acid and acetic acid.

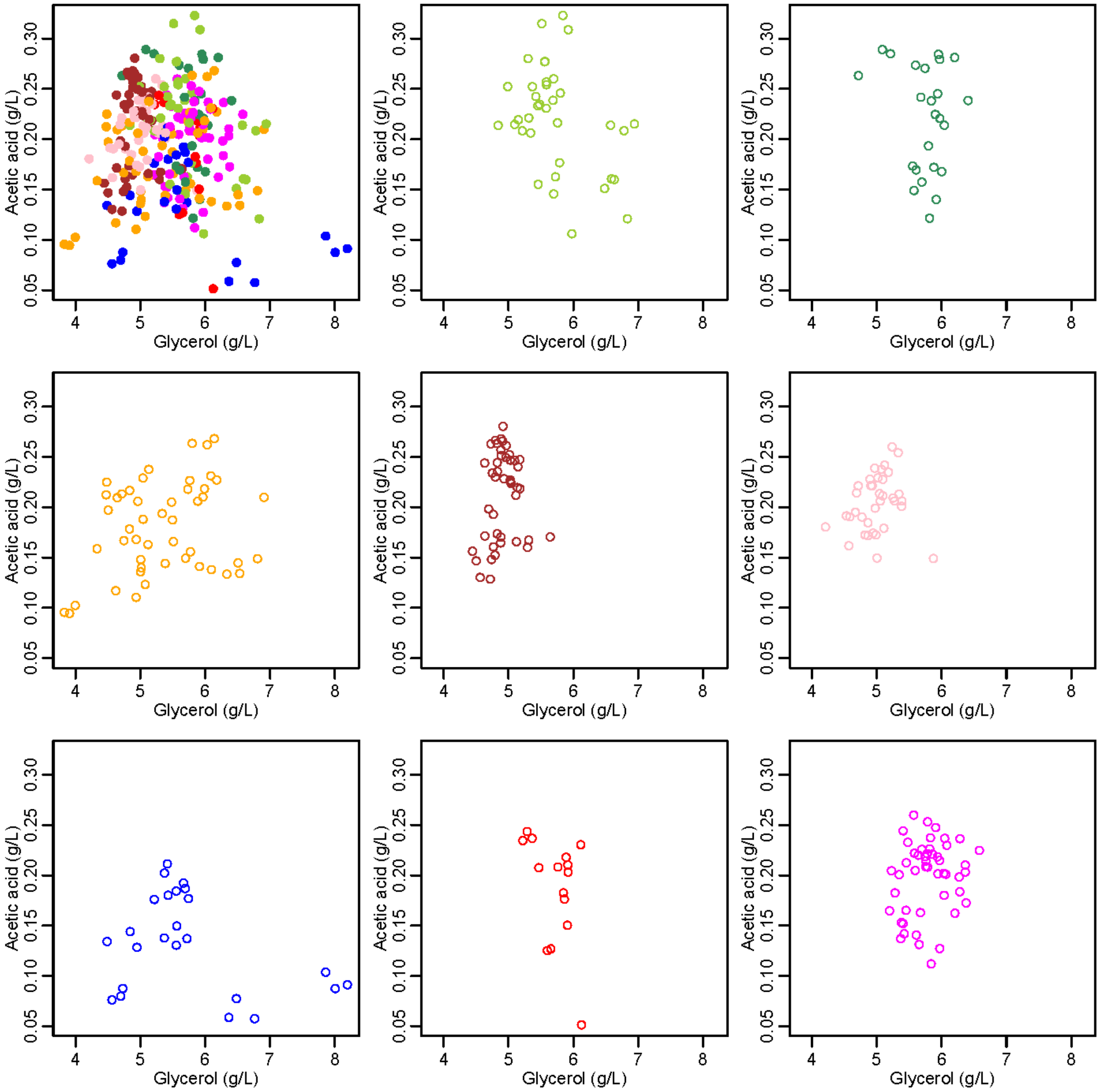

Supplementary Figure 14. Spearman's correlation test between the selected metabolites: glycerol and acetic acid.
